# Transcriptomic analysis predicts the risk of progression of premalignant lesions in human tongue

**DOI:** 10.1007/s12672-023-00629-y

**Published:** 2023-02-23

**Authors:** Tuo Zhang, David Kutler, Theresa Scognamiglio, Lorraine J. Gudas, Xiao-Han Tang

**Affiliations:** 1 Department of Pharmacology, Weill Cornell Medical College of Cornell University, 1300 York Avenue, New York, NY 10065 USA; 2 Division of Head and Neck Surgery in the Department of Otolaryngology at New York Presbyterian Hospital/Weill Cornell Medical Center, New York, NY 10065 USA; 3 Genomics Resources Core Facility, Weill Cornell Medical College of Cornell University, New York, NY 10065 USA; 4 Division of Anatomic Pathology, New York Presbyterian Hospital, Department of Pathology and Laboratory Medicine, Weill Cornell Medical College of Cornell University, New York, NY 10065 USA

**Keywords:** Tongue squamous cell carcinomas, Oral potentially malignant disorders, RNA-seq, Firth logistic regression

## Abstract

The 5-year survival rate for patients with oral squamous cell carcinomas (SCC), including tongue SCC, has not significantly improved over the last several decades. Oral potentially malignant disorders (OPMD), including oral dysplasias, are oral epithelial disorders that can develop into oral SCCs. To identify molecular characteristics that might predict conversion of OPMDs to SCCs and guide treatment plans, we performed global transcriptomic analysis of human tongue OPMD (n = 9) and tongue SCC (n = 11) samples with paired normal margin tissue from patients treated at Weill Cornell Medicine. Compared to margin tissue, SCCs showed more transcript changes than OPMDs. OPMDs and SCCs shared some altered transcripts, but these changes were generally greater in SCCs than OPMDs. Both OPMDs and SCCs showed altered signaling pathways related to cell migration, basement membrane disruption, and metastasis. We suggest that OPMDs are on the path toward malignant transformation. Based on patterns of gene expression, both OPMD and tongue SCC samples can be categorized into subclasses (mesenchymal, classical, basal, and atypical) similar to those seen in human head and neck SCC (HNSCC). These subclasses of OPMDs have the potential to be used to stratify patient prognoses and therapeutic options for tongue OPMDs. Lastly, we identified a gene set (ELF5; RPTN; IGSF10; CRMP1; HTR3A) whose transcript changes have the power to classify OPMDs and SCCs and developed a Firth logistic regression model using the changes in these transcripts relative to paired normal tissue to validate pathological diagnosis and potentially predict the likelihood of an OPMD developing into SCC, as data sets become available.

**Supplementary Information:**

The online version contains supplementary material available at 10.1007/s12672-023-00629-y.

## Introduction

The American Cancer Society estimates 34,730 new oral cancer diagnoses in 2022, including 17,860 new tongue cancer cases within the United States [1]. More than 90% of oral cancer cases are squamous cell carcinomas (SCCs), a type of head and neck SCC (HNSCC) [2, 3]. The 5-year survival rate for patients with oral SCC (OSCC) has not significantly improved, despite various new treatment options, in the last several decades [4]. Current therapies for the majority of oral SCC patients include surgery, cytotoxic chemotherapy, and radiation therapy [2]. Even after treatment about half of the patients relapse, with a subsequent median survival time to 8–10 months [2, 5]. Oral potentially malignant disorders (OPMDs) are a group of oral epithelial disorders that has an increased risk of developing into OSCCs as compared to clinically normal oral mucosa [6]. OPMDs include leukoplakia, erythroplakia, erythroleukoplakia, oral lichen planus, oral submucous fibrosis, and oral dysplasia [7, 8]. An objective system that would predict the tendency of OPMD to develop into OSCC could be helpful both to manage clinical care and to understand the progression of OPMDs to OSCCs.

The current clinical staging system of oral SCCs that determines the basic characteristics and prognosis for patients is based on assessments of primary tumors (Tx, T0, T1, T2, T3, T4a, and T4b), regional lymph nodal metastases (Nx, N0, N1, N2a, N2b, N2c, and N3), and distant metastasis (Mx, M0, M1) [9]. Although this system is useful for determining clinical treatments for OSCC, this arbitrary staging system is not informative in terms of predicting SCC risks for individual patients with OPMDs [10–12]. Therefore, in addition to histopathological assessment for OPMDs, it is important to identify molecular markers that can predict the risks of OPMDs advancing to SCCs and guide treatment plans. These markers could also be very useful in human populations with a high risk of oral cancer, such as people who smoke and/or drink heavily [13].

There have been studies to identify markers to distinguish OPMD and malignant oral lesions [10, 14, 15], however, these studies did not assess global transcript levels during multi-step OSCC carcinogenesis. Researchers have used genome-wide transcriptomics analyses to assess cancer risks, to distinguish human oral leukoplakia subtypes (low and high grade dysplasia) [16–18], to assess OSCCs [19–25], and to predict the clinical outcome of OPMD [26]. Because the patient samples analyzed in these studies [16–26] were obtained from a variety of sites in the human oral cavity, including the tongue, palate, lower/upper gingiva, floor of mouth, buccal mucosa, and sinus, the results may not reflect the genome-wide RNA profiles at specific sites, such as the tongue. Additionally, most of these earlier studies [16–21] used cDNA microarray technology, which has now been replaced by RNA-seq technology because it has a lower background noise, higher specificity, greater dynamic range for quantifying gene expression levels, and the ability to distinguish different transcript isoforms [27]. Although there are studies using RNA-seq [28–30] to characterize human HNSCC, including oral SCC, and identify different molecular events at pathological stages, there are not many studies focusing on tongue OPMD and SCC, a subtype of head and neck cancer. One recent study [22] using RNA-seq technology on brush biopsies of human oral cancers does not have the capacity to show the changes in genome-wide mRNA levels in human OSCC, especially in invasive tumors. Other studies that used RNA-seq focused on miRNA and long non-coding RNAs in human oral cancers [23–25].

Because histopathological characteristics of OPMD do not always accurately predict the clinical outcomes of these lesions, we sought to develop a signature gene set in which transcript level changes in an individual patient with tongue OPMDs could be used to predict the risk of developing SCC. Comparative analysis of samples between OPMDs and SCC is a major challenge because of the variabilities among individual patient transcript changes in these lesions relative to normal tissue samples from the same patient [22]. This variability could be a major reason that there have been few successes in using RNA-based diagnosis or prognosis for oral cancer since many prior studies [10, 14, 19, 21, 30] grouped all normal and lesion samples separately for comparison.

In this report we conducted genome-wide RNA-seq analyses of human tongue OPMDs and SCCs from individual patients compared to the normal healthy tongue epithelia in the same patient. We then developed a gene set that informs the risk of lesions advancing to SCCs. The results from this comparison provide novel molecular markers that may predict the risk of OPMDs becoming OSCCs and have the potential to improve early diagnosis and treatment decisions for OSCCs.

## Materials and Methods

### Human OPMD and SCC samples

Human patient tongue lesion samples and their corresponding margin samples (far away from the lesions) were surgically resected (See Supplementary Information).

### Pathological diagnosis

Histopathologic examination was performed for all lesions (See Supplementary Information).

### RNA-seq analysis of transcriptome

We prepared total RNA from the human margin, OPMD, and SCC samples. Subsequent steps for RNA-seq were carried out at the Genomics Resources Core Facility of WCMC (See Supplementary Information).

### Pathway and gene ontology analysis

We performed pathway and gene ontology analysis using Enrichr (See Supplementary Information).

### SCC subclass correlation studies

We carried out Pearson and Spearman correlation studies using GraphPad Prism software (See Supplementary Information).

### Firth logistic regression analysis

We built a logistic regression model to classify OPMD and SCC samples based on gene expression changes (See Supplementary Information).

### Data and code availability

The data reported in this paper have been deposited in the Gene Expression Omnibus (GEO) database. A web application to predict the SCC risk of a tongue OPMD is available at https://freshtuo.shinyapps.io/sccpred/ (See Supporting Information).

## Results

### Gene expression profiles of human tongue lesions at different pathological stages indicate transcripts involved in human oral SCC progression

**Fig. 1:**
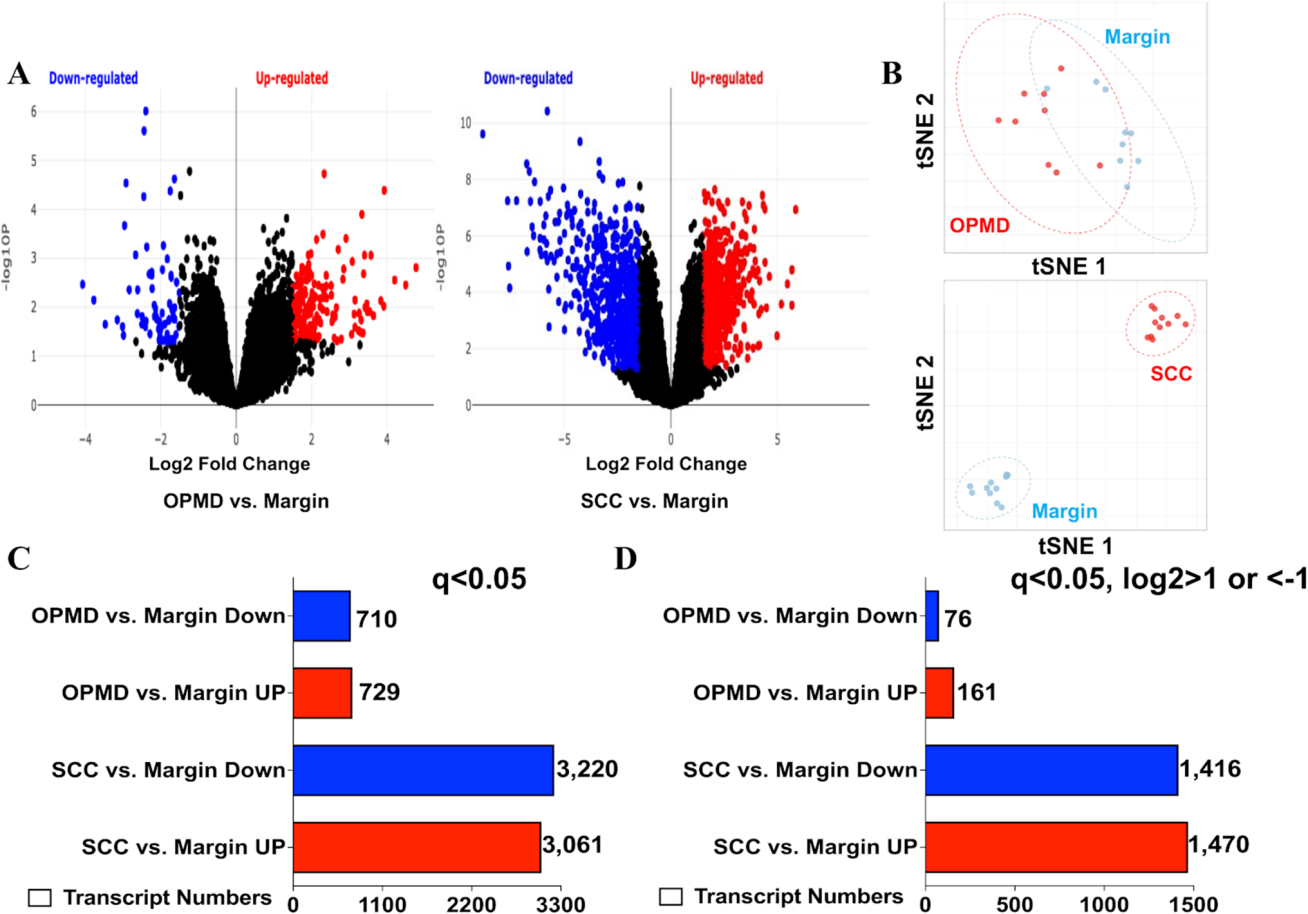
Comparison of transcriptomes between human tongue oral potentially malignant disorders (OPMD), squamous cell carcinoma (SCC), and margins. **A** Volcano plot showing total numbers of transcripts with statistically significant increases or decreases (q < 0.05) in the OPMD (n = 9) and SCC (n = 12) compared with the margin group. **B** tSNE (t-distributed stochastic neighbor embedding plot showing that OPMD is an intermediate state between margin (healthy state) and SCC (cancer state). **C** Numbers of transcripts significantly (q < 0.05) altered OPMD and SCC, vs. margin. **D** Numbers of transcripts significantly (q < 0.05) altered OPMD and SCC, vs. margin, with log2 changes > 1 or < − 1

We performed RNA-seq analysis on the OPMD and SCC samples and the corresponding margin (normal) samples from the same patients. Our analysis revealed that 1439 transcript levels (differentially expressed genes (DEGs)) were altered significantly in the tongue OPMD samples (n = 9) compared to the corresponding margin samples (n = 9), including increases in 729 and decreases in 710 transcripts (adjusted p-value < 0.05) (Fig. 1A, C, Table S1). We identified a total of 6281 transcripts, including 3061 transcripts increased and 3220 transcripts decreased significantly (adjusted p-value < 0.05), in tongue SCC samples (n = 11) compared to the corresponding margin samples (n = 11) (Fig. 1A, C, Table S1). Fold changes in 161 transcripts increased and 76 transcripts decreased were |Log2|> 1, respectively, in OPMD samples vs margin (normal). In SCC samples, 1470 increased and 1416 decreased transcripts showed fold changes of |Log2|> 1, respectively, compared to margin (Fig. 1D). Analysis of transcriptome profiling similarities using a tSNE (t-distributed stochastic neighbor embedding) plot (Fig. 1B) shows that the margin and SCC groups were well separated; samples from each group formed a unique cluster, and in the OPMD group one-third of the samples (n = 3) were mixed with the margin group and two-thirds of the samples (n = 6) were close to the margin group (Fig. 1B). These data suggest that OPMD is an intermediate state between margin (healthy state) and SCC (cancer state).

We analyzed the relationship between the transcripts changed in SCC/margin and that in OPMD/margin with fold changes of |Log2|> 1. A Venn diagram (Figure S1A) shows that there were 146 transcripts that overlapped between SCC/margin and OPMD/margin (Table S2). These transcript overlaps were much more significant than expected by random chance, with a p-value = 3.245 e-73 using Fisher’s exact test (Figure S1B). The log2 fold changes of these 146 transcripts are shown (Figure S1C). Although 141 of these 146 transcripts showed same change directions in SCC/margin and OPMD/margin, with the average fold changes in SCC/margin greater than that in OPMD/margin (Figure S1C), the transcript level changes of these 146 genes in individual patients exhibited variability (Figure S1D). For the following studies below, we only used transcripts with fold changes of |Log2|> 1.

**Fig. 2:**
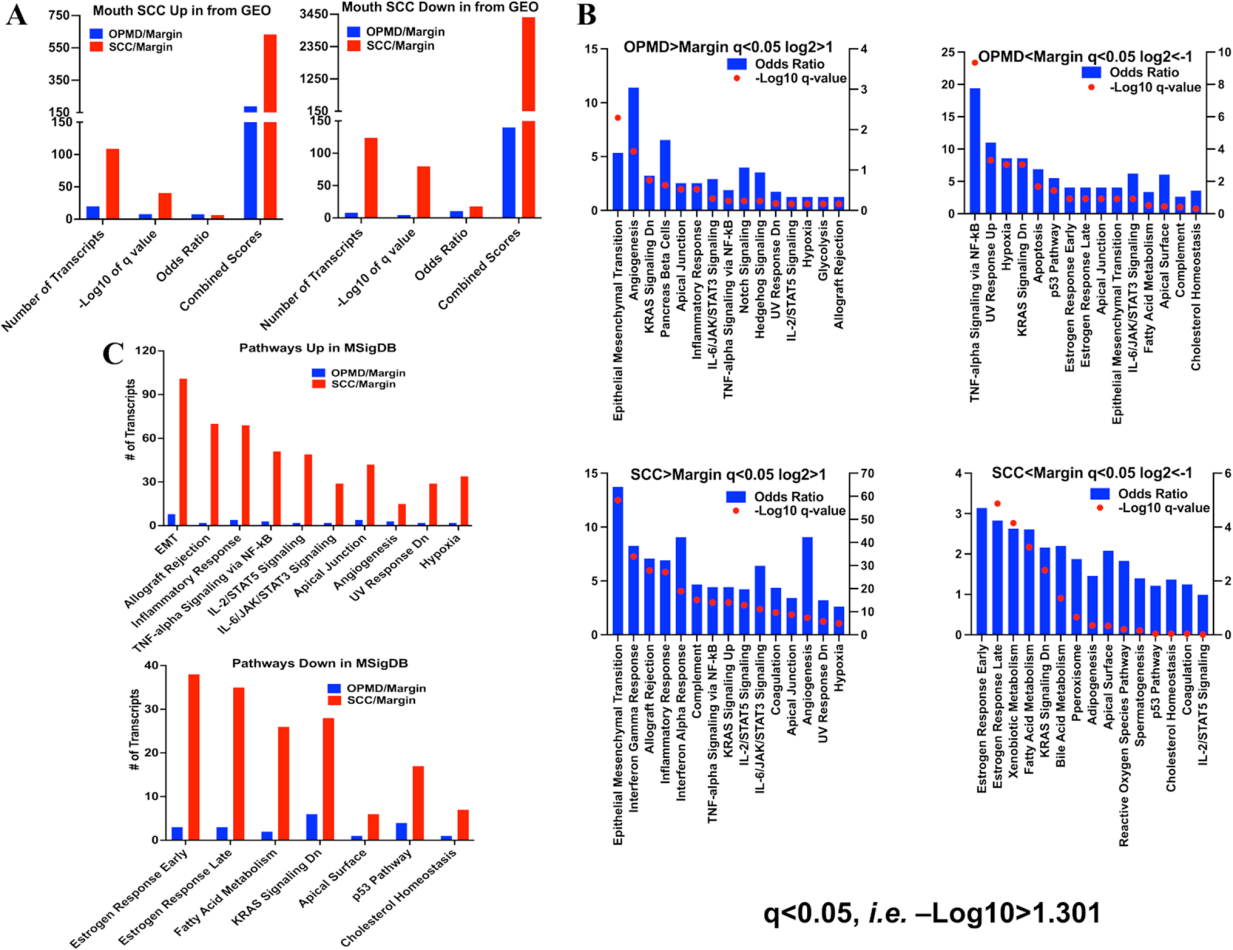
Pathway analysis transcriptomic changes in human tongue oral potentially malignant disorders (OPMD) and squamous cell carcinoma (SCC). **A** The association between significantly altered transcripts (q < 0.05, log2 > 1 or < − 1) in OPMD and SCC and human oral SCC using Disease Perturbations from GEO database. **B** Pathway enrichment analysis of significantly altered transcripts (q < 0.05, log2 > 1 or < − 1) in OPMD and SCC using Molecular Signatures Database (MSigDB). Left Y axis, odds ratio; right Y axis, -Log10 q-value. **(C)** The numbers of altered transcripts (q < 0.05, log2 > 1 or < − 1) in OPMD and SCC enriched in the top 15 pathways derived from MSigDB analysis

 To ascertain whether our patients’ lesions captured the characteristics of human oral SCC, we performed disease signature pathway analysis using “Disease Perturbations from GEO up/down” [31], a platform designed to probe a variety of gene and disease associations. This disease association study verified that both OPMD and SCC samples closely resembled human oral SCC molecular features (Table S3). SCC samples exhibited more human oral SCC transcript markers than OPMD samples, based on the numbers of transcripts, q values, odds ratios, and combined scores (Fig. 2A), indicating that SCC is a more advanced pathologic state than OPMD.

### Transcripts altered in pathways in human tongue lesions at different stages are associated with pathological stages of the tongue lesions

We conducted a pathway enrichment analysis of the transcripts altered in the OPMD and SCC samples using the Enrichr web tool, based on the MsigDB (Molecular Signatures Database) [32]. The top 15 pathways enriched in OPMD and SCC samples are shown (Fig. 2B). In OPMD samples, only pathways “Epithelial Mesenchymal Transition” (EMT) and “Angiogenesis”, enriched with increased transcript levels, were statistically significant (q < 0.05), while in SCC samples, all top 15 pathways enriched with elevated transcript levels were statistically significant, including “Epithelial Mesenchymal Transition” and “Angiogenesis” (Fig. 2B, Table S4). The top 6 pathways enriched with decreased transcript levels in both OPMD and SCC samples were statistically significant (Fig. 2B, Table S4). We then compared the numbers of increased and decreased transcripts in the top overlapped pathways between OPMD and SCC groups (Fig. 2C). Within the 10 and 7 overlapped pathways enriched with increased and decreased transcripts levels, respectively, SCC samples showed changes in more transcripts than OPMD samples. These data, along with the greater average fold changes in SCC/margin than in OPMD/margin (Figure S1C), indicate that SCC shows more intense perturbations on these pathways than OPMD. Importantly, the “Epithelial Mesenchymal Transition” and “Angiogenesis” pathways (Fig. 2B, C) suggest that cancer cell migration and metastasis occur at the pre-SCC stage of human tongue carcinogenesis.

### Transcripts altered in pathways in human tongue lesions at different stages are associated with pathological stages of the tongue lesions

By using the KEGG (Kyoto Encyclopedia of Genes and Genomes) pathway analysis program we discovered that the top 15 pathways significantly enriched with transcripts increased in the SCC group included the cancer-related pathways “Cytokine-cytokine receptor interaction” and “ECM-receptor interaction,” while there were no cancer-related pathways with increased transcripts significantly (q < 0.05) enriched in the OPMD group (Figure S2, Table S5). In the SCC group with transcripts decreased “Metabolism of xenobiotics by cytochrome P450” and “Fatty acid degradation” were among the top 8 pathways significantly (q < 0.05) enriched. The top pathways significantly (q < 0.05) enriched with transcripts decreased in the OPMD group, such as “Estrogen signaling pathway” and “Lipid and atherosclerosis,” showed no directly cancer-related pathways (Figure S2, Table S5). Thus, our KEGG pathway analysis shows that cancer-related pathways are more highly activated in the SCC than in OPMD samples.

Gene set enrichment analysis (GSEA) using the Gene Ontology (GO) Biological Process Database revealed that the top 15 pathways with transcripts increased in the SCC group included “Extracellular matrix organization (GO:0030198)” and “Inflammatory response (GO:0006954)”, and the top pathways enriched with transcripts in the OPMD group showed “Skin development (GO:0043588)” and “Extracellular matrix organization (GO:0030198)” (Figure S3, Table S5). Additionally, the top pathways with transcripts decreased in the SCC and OPMD groups included “Epidermis development (GO:0008544)” and “Cellular heat acclimation (GO:0070370)” (Figure S3, Table S5). The KEGG and GO pathways enriched with transcripts increased in the OPMD and SCC samples suggest: (1) an increase in cell motility potential in SCC and OPMD [9], with greater potential in SCC than OPMD, in line with the data from the MSigDB analysis (Fig. 2B); and (2) perturbation of the immune environment in SCC, *e.g.* increases in interferon responses and cytokine signaling (Fig. 2B).

### The changes in gene expression profiles in individual patients categorize these OPMD and SCC lesions into distinct subclasses

**Fig. 3:**
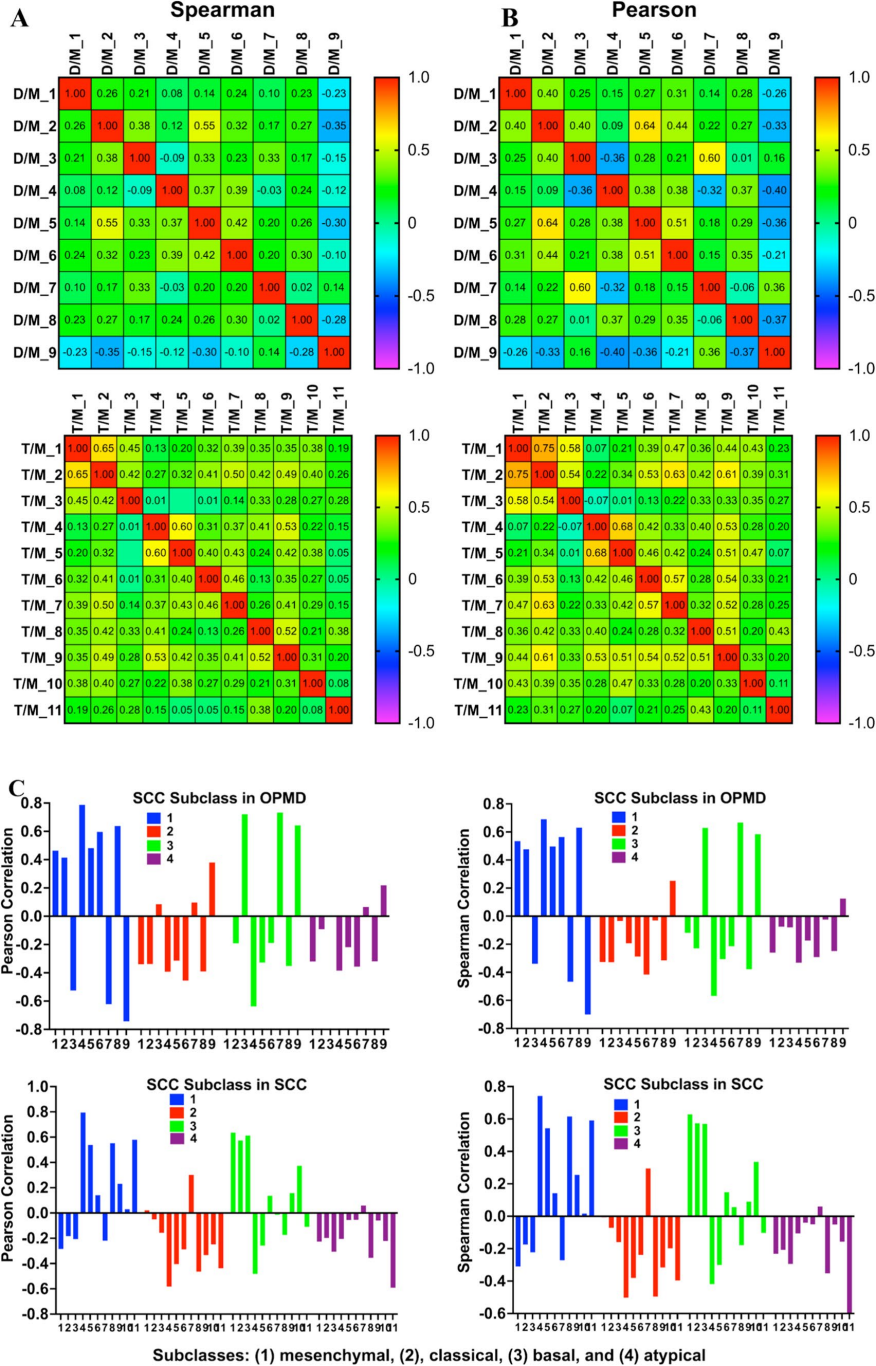
Correlation studies of the variabilities among the global transcriptomic changes (q < 0.05, log2 > 1 or < − 1) in individual OPMD or SCC, compared to the margin from the same patient. **A** Spearman and **B** Pearson correlation analyses of the variabilities and association among the OPMD and SCC samples. **C** Human head and neck squamous cell carcinoma (SCC) subclass categorization of the OPMD and SCC samples, based on the global transcriptomic changes (q < 0.05, log2 > 1 or < − 1) in individual OPMD or SCC. D (disorders), OPMD; M, margin; T (tumor), SCC; D/M, OPMD/margin; T/M, SCC/margin

A global heatmap analysis on the RNA-seq data, with each margin sample followed by the lesion sample from the same patient (OPMD or SCC) (Figure S4) demonstrated that there were differences in the basal gene expression in the margins in different patients. It was also clear that there were differences between the margin tissue and the OPMD or SCC samples. Furthermore, the changes in the average levels of individual transcripts in the tongue OPMD and SCC groups vs. the margin groups did not always reflect the changes in individual patients (Table S2, Figure S1C and D, as an example). These data indicate divergent gene expression in human tongue OPMD and SCC samples as well as variations in gene expression in normal tissues. Therefore, we next compared the changes in the transcriptomes in individual tongue lesion vs. margin tissue from the same patient. This comparison could mitigate the possible errors caused by comparing the group average of all margin samples with that of all lesion samples and reveal more consistent changes between OPMD and OSCC. We performed correlation studies (Spearman and Pearson) using the log2 fold changes of transcripts between tongue lesions (OPMD or SCC) vs. their margins in the same patients to examine the similarities in the entire transcriptome among patients. In the OPMD group, samples #5 and #2 were quite similar, and sample #9 showed no correlations with all other samples except a small similarity to #7 (Fig. 3A), shown by both Pearson and Spearman correlation studies. In the SCC group, samples #1, and #2; #3, #4 and #5; and #8 and #9 showed higher similarities (r > 0.5) than other sample comparisons using both Spearman and Pearson correlations (Fig. 3A). These data confirm variability by varied transcript level changes among human tongue lesions, including OPMD and SCC.

Previous researchers have discovered four distinct, clinically relevant subclasses of human head and neck SCC based on the gene expression patterns: [1] mesenchymal, [2], classical, [3] basal, and [4] atypical [33, 34]. We next examined whether our individual human tongue OPMD and SCC samples could be categorized into these subclasses, based on the transcriptomic changes in an individual tongue lesion vs. margin tissue from the same patient. We performed correlation study using the transcriptomic changes (|Log2|> 1) in each patient (Table S6) and the mRNA markers for each subclass of human head and neck SCC downloaded from the Broad Firehose website (https://gdac.broadinstitute.org/). This study (Fig. 3C) shows that in the OPMD group samples #1, 2, 4, 5, 6, and 8 correlated with subclass 1 (mesenchymal); #3 and 7 correlated with subclass 3, (basal); and #9 correlated with both subclasses 2 (classical) and 3 (basal), indicating that #9 showed features of these two subclasses. In the SCC group samples #1, 2, 3, and 10 correlated with subclass 3 (basal); samples #4, 5, 8, and 11 correlated with subclass 1 (mesenchymal); sample #7 correlated with subclass 2 (classical); and samples #6, 8 did not show a strong correlation with any subclasses (Fig. 3C). The correlations between samples in the OPMD and SCC groups, based on the mRNA markers for each human HNSCC subclass, are shown (Figure S5); this extends data in Fig. 3C. Notably, most of these lesions belonged to the mesenchymal and basal subclasses, while for both OPMD and SCC groups the classical and atypical subclasses were rare.

### Firth logistic regression analysis shows the transcript changes in individual patients that differ between OPMD and SCC

Because the majority of OPMD do not progress to SCC [6, 26], we develop an machine learning approach to classify OPMDs and SCCs using a signature gene set whose transcript changes in a patient’s tongue lesion, compared to the same patient’s margin tissue, and predict the probability of a lesion to progress to SCC. We implemented our analysis using Firth logistical regression to alleviate possible overfitting caused by small sample size [35]. We performed the analysis on the 6,693 transcripts selected by taking a union of the differential expressed genes (adjusted p-value < 0.05) derived from the two groups (OPMD and SCC) and ranking the transcripts by their area under curve (AUC) scores of the Receiver Operator Characteristic (ROC) curve, a graphical plot illustrating the diagnostic ability of a binary classifier system as its discrimination threshold is varied (Table S7). The AUC values of 848 and 149 transcripts were above 0.8 and 0.9, respectively, indicating sufficient power to distinguish OPMD from SCC (Table S7). Then we applied four filters (described in MATERIALS AND METHODS, Supplementary Information) to refine candidate transcript selections.

**Fig. 4:**
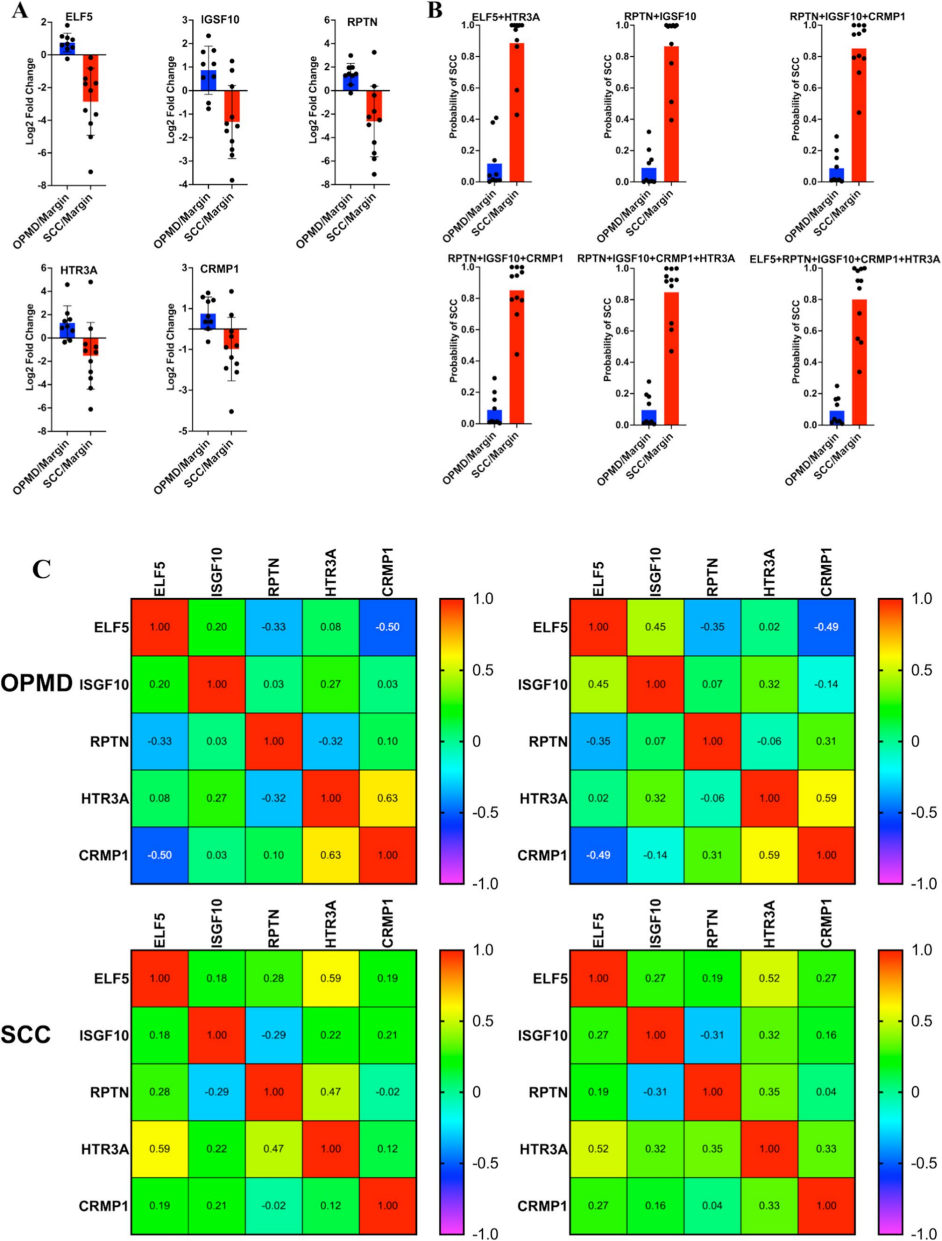
Transcript level changes of ELF5, RPTN, IGSF10, HTR3A, and CRMP1 in individual OPMD and SCC samples and correlation studies among them. **A** Transcript level changes of these transcripts in individual patients. **B** The probabilities of SCC of individual tongue lesion samples derived from Leave-One-Out Test”. **C** Pearson and Spearman correlation studies among ELF5, RPTN, IGSF10, CRMP1, and HTR3A transcripts within the OPMD and SCC groups

We screened five candidate transcripts as described in the Materials and Methods: ELF5 (E74 Like ETS Transcription Factor 5), IGSF10 (Immunoglobulin Superfamily Member 10), CRMP1 (Collapsin Response Mediator Protein 1), RPTN (Repetin), and HTR3A (5-Hydroxytryptamine Receptor 3A) (Table S6). ELF5 and IGSF10 mRNA levels are significantly decreased in human head and neck cancers [36, 37]. CRMP1 inhibits prostate cancer cell migration and metastasis by suppressing EMT [38]. Although there are no reports of HTR3A in human tongue SCC, elevated HTR3A expression correlates with increased human lung adenocarcinoma cell proliferation and is associated with aggressive histopathology [39]. RPTN is associated with epithelial differentiation [40]. The log2 changes of these transcripts in each patient’s tongue lesion vs. the margin tissue are shown (Fig. 4A). The average changes in these transcript levels in tongue SCC were in line with previous studies described above. Importantly, we show that the average changes of these transcripts in OPMD and SCC occurred in opposite directions, which underscores the potential of the changes in these transcripts to differ OPMDs from SCCs.

We further tested all transcript combinations and their power to distinguish OPMD from SCC, out of which 6 combinations had the highest power (AUC = 1) with “Leave-One-Out” cross-validation (Table S7). The results for individual samples are shown (Fig. 4B and Table S8), indicating that the combinations of these transcript changes have the potential to separate OPMD and SCC samples.

Transcripts whose changes are well correlated are not considered independent predictors of outcome [41]. We evaluated if there was a strong correlation between any two of the five potential markers. Correlation analysis shows no significant correlations in both OPMD and SCC groups (p > 0.05) (Fig. 4C and Table S9). Because there is no database specific about tongue SCC, a subtype of HNSCC, we then used the HNSCC TCGA Pan-Cancer Atlas database (https://www.cbioportal.org/) to validate the correlations between the changes of these 5 transcripts in SCC relative to margin. Neither CRMP1 (Figure S6A-D) nor IGSF10 (Figure S6D-G) were correlated with the other 4 markers. ELF5, HTR3A, and RPTN were weakly correlated with each other (Figure S6H-J). Thus, changes in these transcripts can serve as independent predictors of outcome.

We then built six SCC prediction models using the six transcript combinations (Table S7) on all samples to predict the risk of an individual patient’s tongue lesion to become SCC. The models are listed below:

$$\begin{array}{*{20}c} {P = 1/(1 + e^{{ - ( - 1.5937842 - 3.0567907*ELF5 + 0.4074382*HTR3A)}} )} & {{\mathbf{p}}{\mkern 1mu} = {\mkern 1mu} {\mathbf{0}}.{\mathbf{00015971}}} \\ \end{array}$$

$$P = 1/(1 + e^{ - ( - 0.5560738 - 1.4883524*RPTN - 1.7171354*IGSF10)} ){\kern 1pt} \quad {\mathbf{p}} = {\mathbf{0}}.{\mathbf{0001055603}}$$

$$P = 1/(1 + e^{ - ( - 0.9167024 - 1.8009372*ELF5 - 0.8139859*CRMP1 + 0.1961076*HTR3A)} )\quad {\mathbf{p}} = {\mathbf{0}}.{\mathbf{000623414}}$$

$$P = 1/(1 + e^{ - ( - 0.7026886 - 0.9277354*RPTN - 0.9976144*ISGF10 - 0.6827067*CRMP1)} )\quad {\mathbf{p}} = {\mathbf{0}}.{\mathbf{0005281575}}$$

$$P = 1/(1 + e^{ - ( - 0.76304119 - 0.79095631*RPTN - 0.98737768*IGSF10 - 0.62807910*CRMP1 + 0.06449884*HTR3A)} )\quad {\mathbf{p}} = {\mathbf{0}}.{\mathbf{0023437143}}$$

$$P = 1/(1 + e^{ - ( - 0.8914550 - 0.1397542*ELF5 - 0.7157705*RPTN - 0.8221783*IGSF10 - 0.5300456*CRMP1 + 0.1286990*HTR3A)} )\quad {\mathbf{p}} = {\mathbf{0}}.{\mathbf{00582664}}$$

We finally chose the gene combination “RPTN, IGSF10” as a diagnostic signature to predict the probability of a OPMD becoming SCC because RPTN and IGSF10 were independent predictors of outcome and had statistically significant correlations with the sample groups (OPMD or SCC), with p-values equal to 1.4E-3 and 2.8E-3, respectively (Table S10). We then applied this model to all lesion samples and calculated their probabilities to become SCC (see Table 1), indicating that our prediction model could be complementary to pathological diagnosis.

**Table 1:** Predicted chance (%) of SCC in our samples using Firth logistic regression model

	RPTN + IGSF10		RPTN + IGSF10
OPD1	1.21	SCC1	99.37
OPD2	0.50	SCC2	100.0
OPD3	0.10	SCC3	76.57
OPD4	0.17	SCC4	73.65
OPD5	0.13	SCC5	90.89
OPD6	22.96	SCC6	99.20
OPD7	14.60	SCC7	99.69
OPD8	8.81	SCC8	81.65
OPD9	10.32	SCC9	100.0
		SCC10	99.14
		SCC11	100.0

The logistic regression model not only distinguishes OPMD from SCC states and validate pathological diagnosis, but also has potential to predict the probability of a sample progressing towards SCC. Therefore, we propose the gene combinations “RPTN and IGSF10” as a diagnostic signature gene set because these transcript changes were independent predictors of outcome. We have prepared an app for physicians to validate pathological assessment and potentially predict SCC risk of a tongue OPMD: https://freshtuo.shinyapps.io/sccpred/. To use these two genes in this model, a physician would do the following: (A) Get RNA-seq of the lesion vs. normal tissue nearby from biopsies; (B) Open the app, input the log2 fold changes in these two transcript levels (lesion vs. normal margin); then press “submit”; (C) See the SCC probability result (See an example in the Appendix). This logistic regression model shows proof of concept that the changes in the transcript levels (lesion vs. margin) has potential to verify pathological diagnosis and potentially predict the SCC risk of a tongue lesion. To validate this prediction, the algorithm should be tested on an independent data set, but currently we do not have such a dataset available.

## Discussion

Tongue OPMDs are lesions with a high risk of progressing to SCC [6]. Our genome-wide gene expression profiling of human tongue OPMD and SCC samples identified changes in the transcripts that are associated with the clinical pathological diagnosis: **(1**) Compared to the margin samples, OPMD samples show changes in much fewer transcripts compared to SCC samples; **(2)** The number of changed transcripts resembling human oral SCC molecular features in the OPMD samples is lower than that in the SCC samples. These data indicate that, as expected, OPMDs display early pathological changes that may lead to SCC development. The paired design allows us to remove variations among individuals so that we can focus on variations between OPMD/SCC and normal conditions.

Multiple overlapping pathway analyses suggest that EMT and cell migration start in OPMD and that higher signals in the SCC samples indicate that in EMT cell migration occurs more frequently. These data suggest that “quasi-normal” epithelial cells start to disseminate from pre-neoplastic lesions at an early carcinogenesis stage, such as OPMD. These results are in line with the “parallel progression model” of metastasis, *i.e*., metastasis is initiated long before the primary tumor is well developed and diagnosed [42, 43]. Notably, clinical studies have shown that oral leukoplakia exhibits basement membrane disruption [44, 45].

We categorized these OPMD and SCC lesions into distinct classes and confirmed the heterogeneity among individual human tongue pre-cancerous and cancerous lesions. Importantly, to our knowledge this is the first report in which, based on the transcriptomic changes in OPMD/margin in individual patients, human OPMD samples are classified into different head and neck SCC subclasses (mesenchymal, basal, classical, and atypical). Previous studies [33, 34, 46–48] suggest that this subclass categorization has clinical relevance and has the potential to serve as a reference for patient prognosis and therapeutic options for tongue SCC, and especially OPMD, so this correlation strengthens the possibility that gene profile analysis could be a useful tool to guide prognosis and therapy.

Various prognostic molecular markers for OPMD, including p53, Ki67 and PCNA, cell cycle proteins, loss of heterozygosity (LOH), and some cell surface and stromal proteins, identified in prior studies, have failed to be used in clinical practice for OPMD prognosis [10, 49] because many studies did not have the adequate follow-up required by the longitudinal design criteria and well-defined diagnostic criteria [49]. Recently, researchers proposed a gene set with 11 genes to predict the risk of OPMD progression to SCC [26]. This research used oral lesion samples, not just tongue lesions, and did not focus on transcript changes in individual patients. Moreover, researchers extracted RNA from formalin-fixed paraffin-embedded (FFPE) tissues, which could produce higher data variation at the single gene level, despite the applicability of FFPE tissue for global gene expression analysis [50]. Thus, our high quality, total RNA samples from fresh human tissue (RIN > 8.5) give a more accurate measurement on a single transcript level, and this is critical in generating a gene set signature.

Here we focused on paired cases of OPMD and SCC, *i.e*., a tongue lesion versus the tongue margin tissue in the same patient. We think that this type of comparison is optimal for addressing the problem of variability in transcript changes among patients. Additionally, we screened candidate transcripts using the opposite directions of fold changes observed in OPMD and SCC groups. Therefore, our prediction model derived from the screened transcripts has the potential to distinguish OPMD from SCC and predict the risks of OPMDs progressing to SCCs. This will help physicians decide whether to surgically remove an OPMD.

One limitation of our study is the relatively small sample size. Thus, validation with an independent data set is needed. Another point is that we have only analyzed mRNA, and not protein levels of these genes. In future work we plan to examine the levels of these proteins in OPMDs. However, we have shown proof of principle that changes in a set of transcripts between a tongue lesion and normal tongue epithelial tissue from the same patient have the potential to classify OPMDs and SCCs and provide insights to the probability of OPMDs becoming oral SCCs.

## Conclusion

We have built a Firth logistic regression model using the changes in these transcripts relative to paired normal tissue to validate pathological diagnosis and potentially predict the likelihood of an OPMD developing into SCC, as data sets become available.

### Supplementary Information

Supplementary file 1 (PDF 6828 KB)

Supplementary file 2 (DOCX 33 KB)

Supplementary file 3 (XLSX 7390 KB)

Supplementary file 4 (XLSX 46 KB)

Supplementary file 5 (XLSX 400 KB)

Supplementary file 6 (XLSX 39 KB)

Supplementary file 7 (XLSX 852 KB)

Supplementary file 8 (XLSX 164 KB)

Supplementary file 9 (XLSX 923 KB)

Supplementary file 10 (XLSX 12 KB)

Supplementary file 11 (XLSX 10 KB)

Supplementary file 12 (XLSX 12 KB)

## Appendix

For example, in one patient, the log2 fold change (lesion vs. margin) in RPTN and IGSF10 transcript levels are 1.5 and 2.5, respectively, then the calculated the SCC risk is 0.084%; in another patient, the log2 fold change (lesion vs. margin) in RPTN and IGSF10 transcript levels are − 0.5 and − 1, respectively, then the calculated the SCC risk is 87.05%. Thus, the second patient has a higher SCC risk than the first patient.
