# Supplementary material for: Transcriptomic analysis predicts the risk of progression of premalignant lesions in human tongue
Source: Discov Oncol. 2023 Feb 23;14:24. doi: 10.1007/s12672-023-00629-y (PMC9950315; doi:10.1007/s12672-023-00629-y)
Supplement: Supplementary file 1 — (PDF 6828 KB) [file 12672_2023_629_MOESM1_ESM.pdf]

A

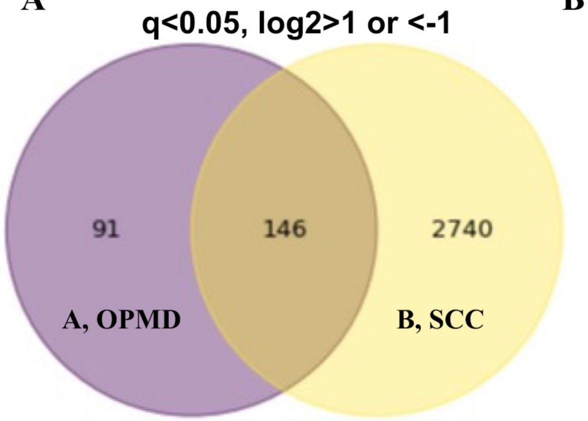

B

Fisher Exact Test

	Not in A	In A	Marginal Row Totals
**Not in B**	20709	91	20800
**In B**	2740	146	2886
**Marginal Column Totals**	23449	237	23686 (Grand Total)

p-value: 3.245e-73

Odds Ratio: 12.126068821689259

Result: This result is **significant** at $p < 0.05$

C

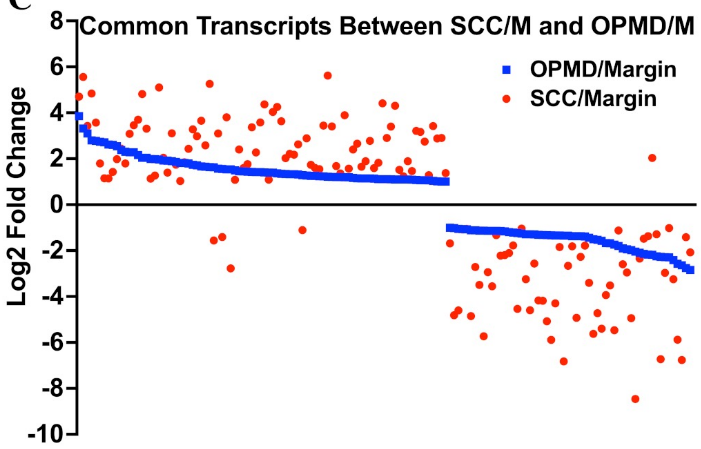

D

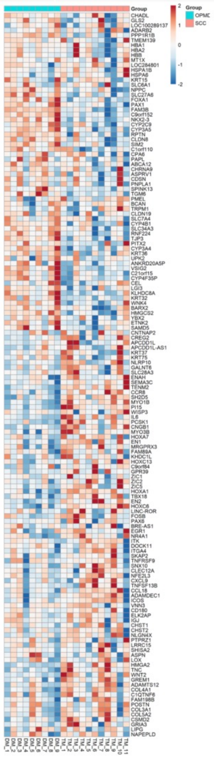

Figure S1.

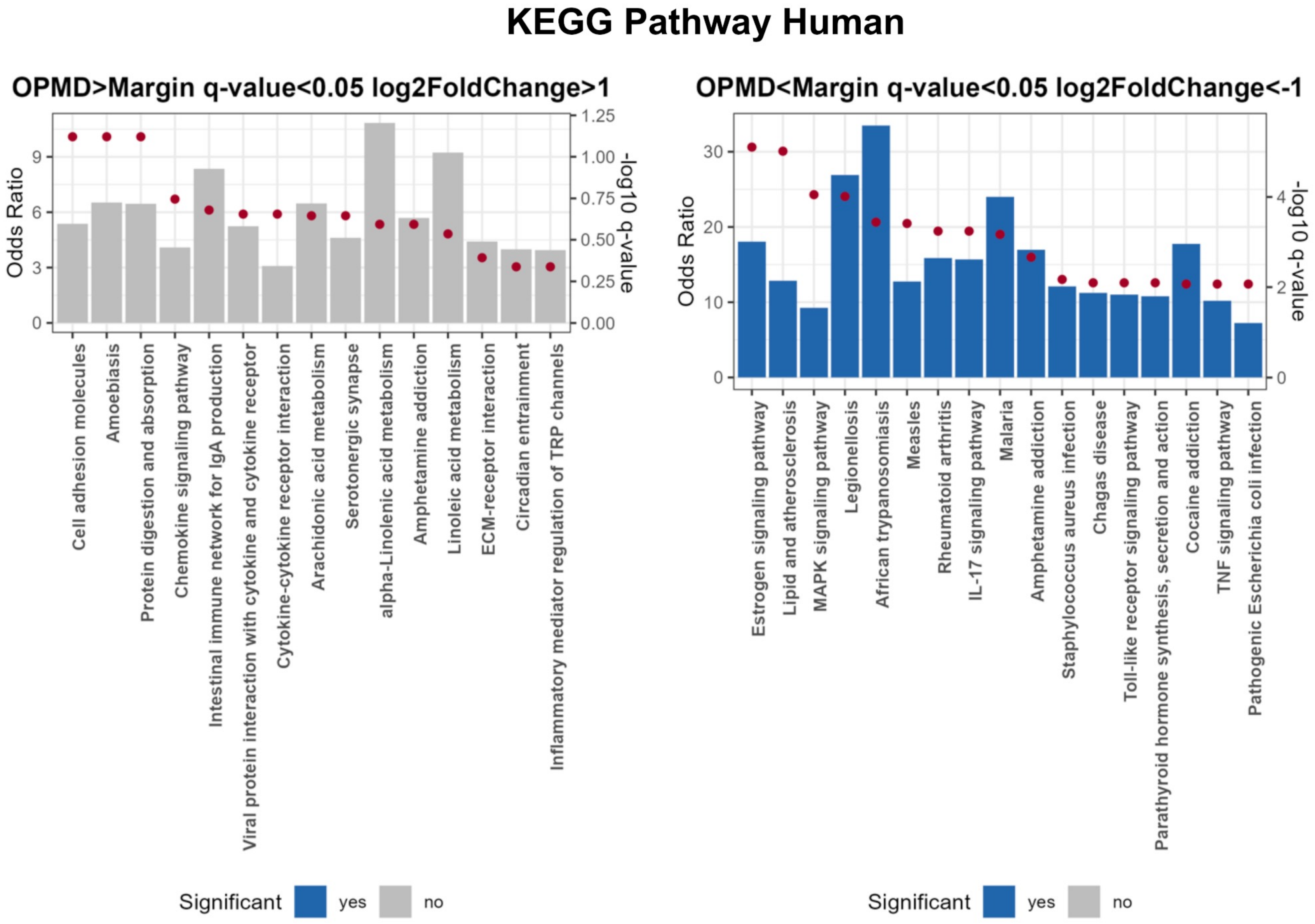
KEGG Pathway Human
OPMD>Margin q-value<0.05 log2FoldChange>1
Odds Ratio
-log10 q-value
0
3
6
9
0.00
0.25
0.50
0.75
1.00
1.25
Cell adhesion molecules
Amoebiasis
Protein digestion and absorption
Chemokine signaling pathway
Intestinal immune network for IgA production
Viral protein interaction with cytokine and cytokine receptor
Cytokine-cytokine receptor interaction
Arachidonic acid metabolism
Serotonergic synapse
alpha-Linolenic acid metabolism
Amphetamine addiction
Linoleic acid metabolism
ECM-receptor interaction
Circadian entrainment
Inflammatory mediator regulation of TRP channels
Significant
yes
no
OPMD<Margin q-value<0.05 log2FoldChange<-1
Odds Ratio
-log10 q-value
0
10
20
30
0
2
4
Estrogen signaling pathway
Lipid and atherosclerosis
MAPK signaling pathway
Legionellosis
African trypanosomiasis
Measles
Rheumatoid arthritis
IL-17 signaling pathway
Malaria
Amphetamine addiction
Staphylococcus aureus infection
Chagas disease
Toll-like receptor signaling pathway
Parathyroid hormone synthesis, secretion and action
Cocaine addiction
TNF signaling pathway
Pathogenic Escherichia coli infection
Significant
yes
no

Figure S2.

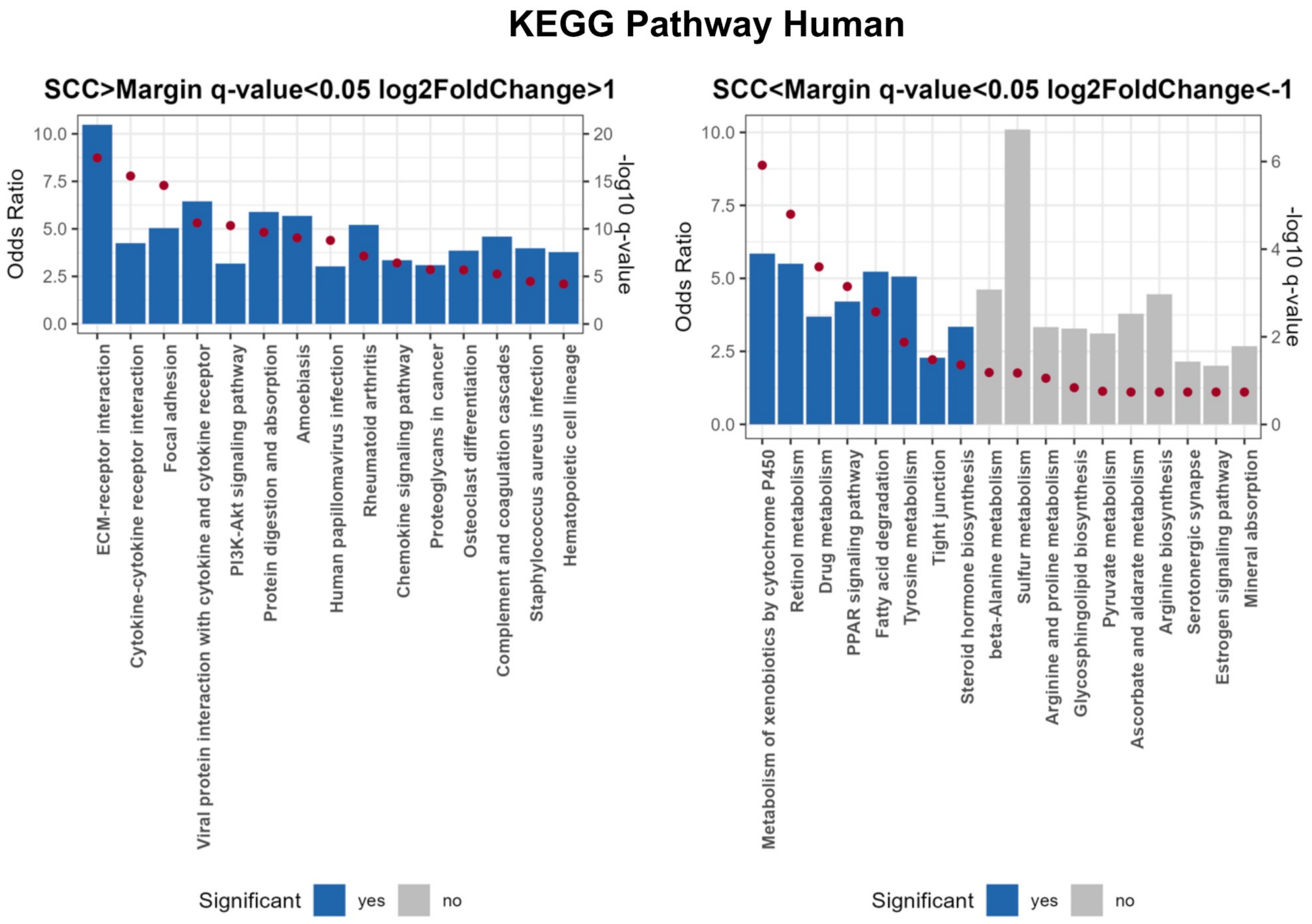

Figure S2 continued.

GOBP Database.

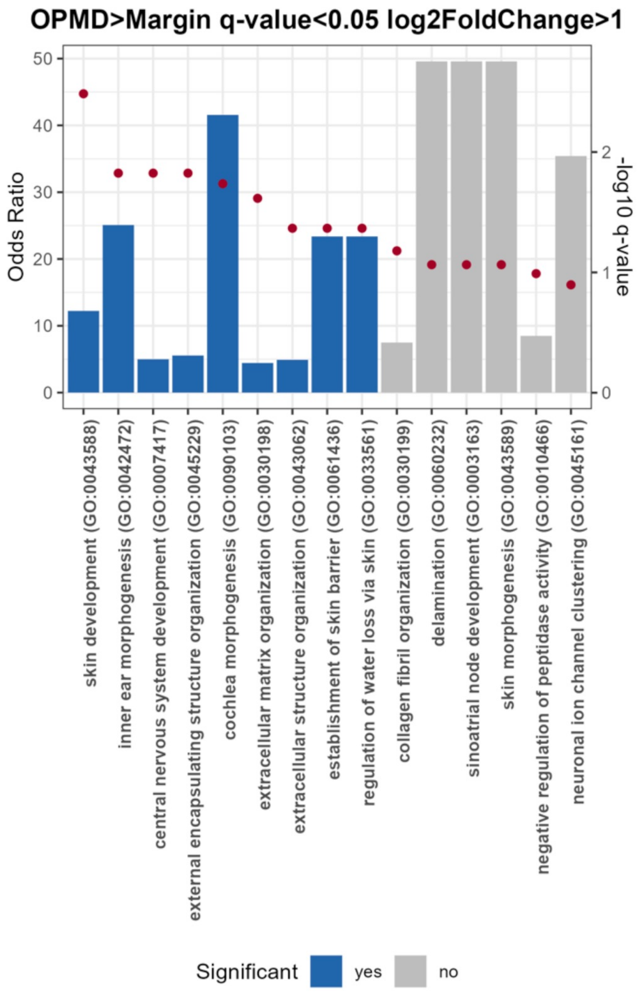

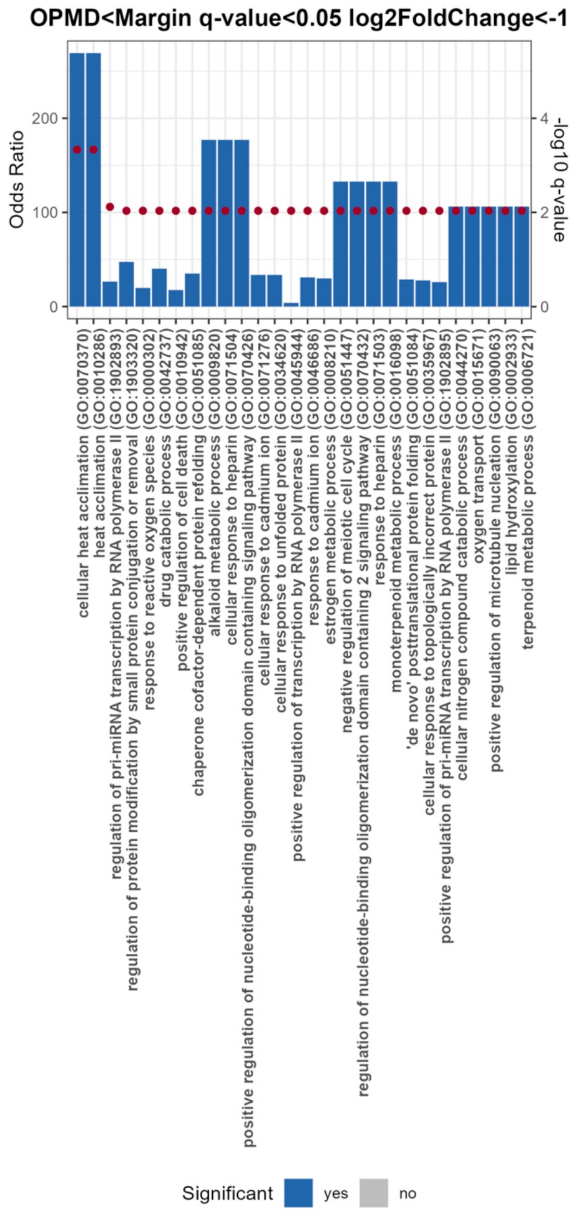

Figure S3.

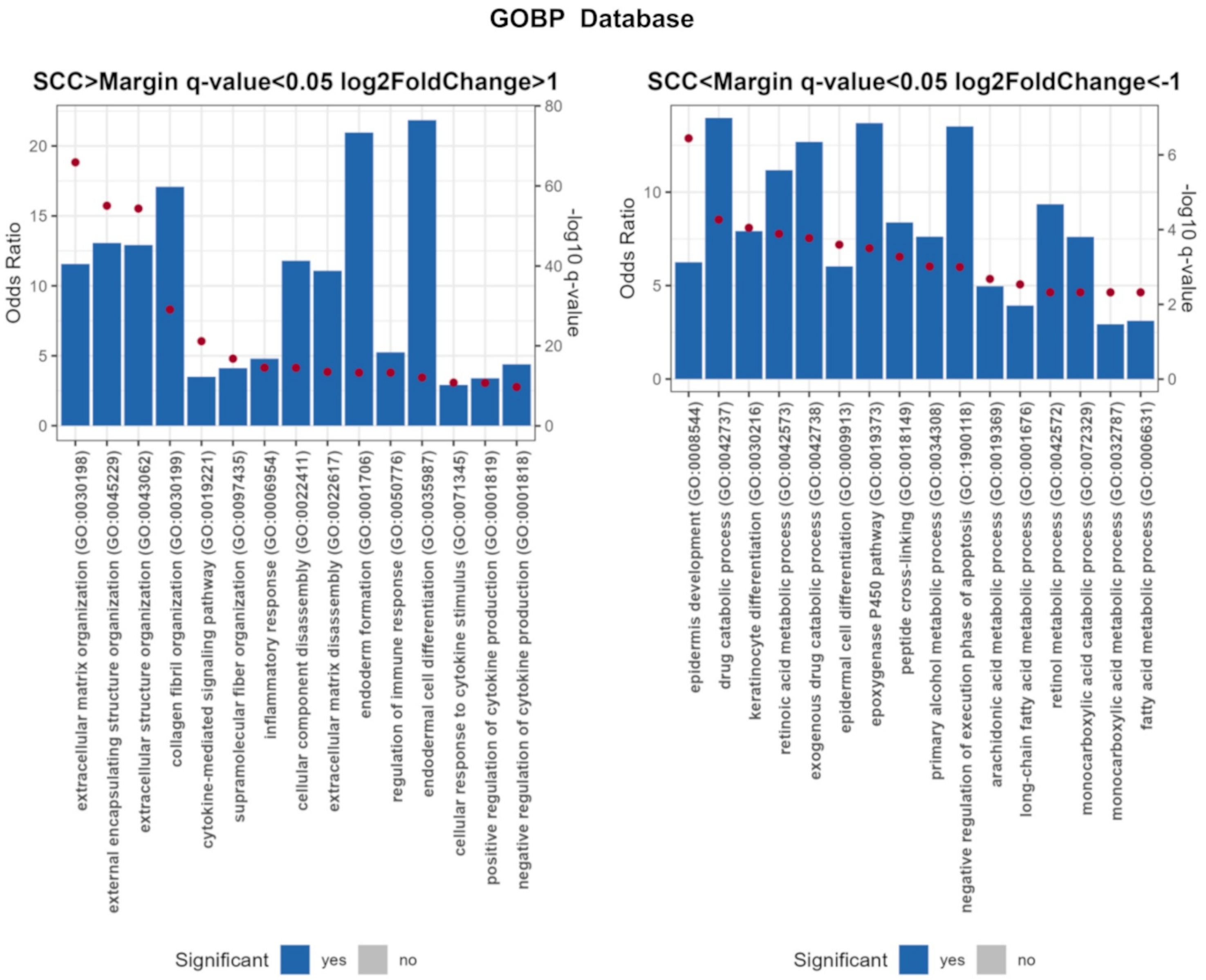

Figure S3 continued.

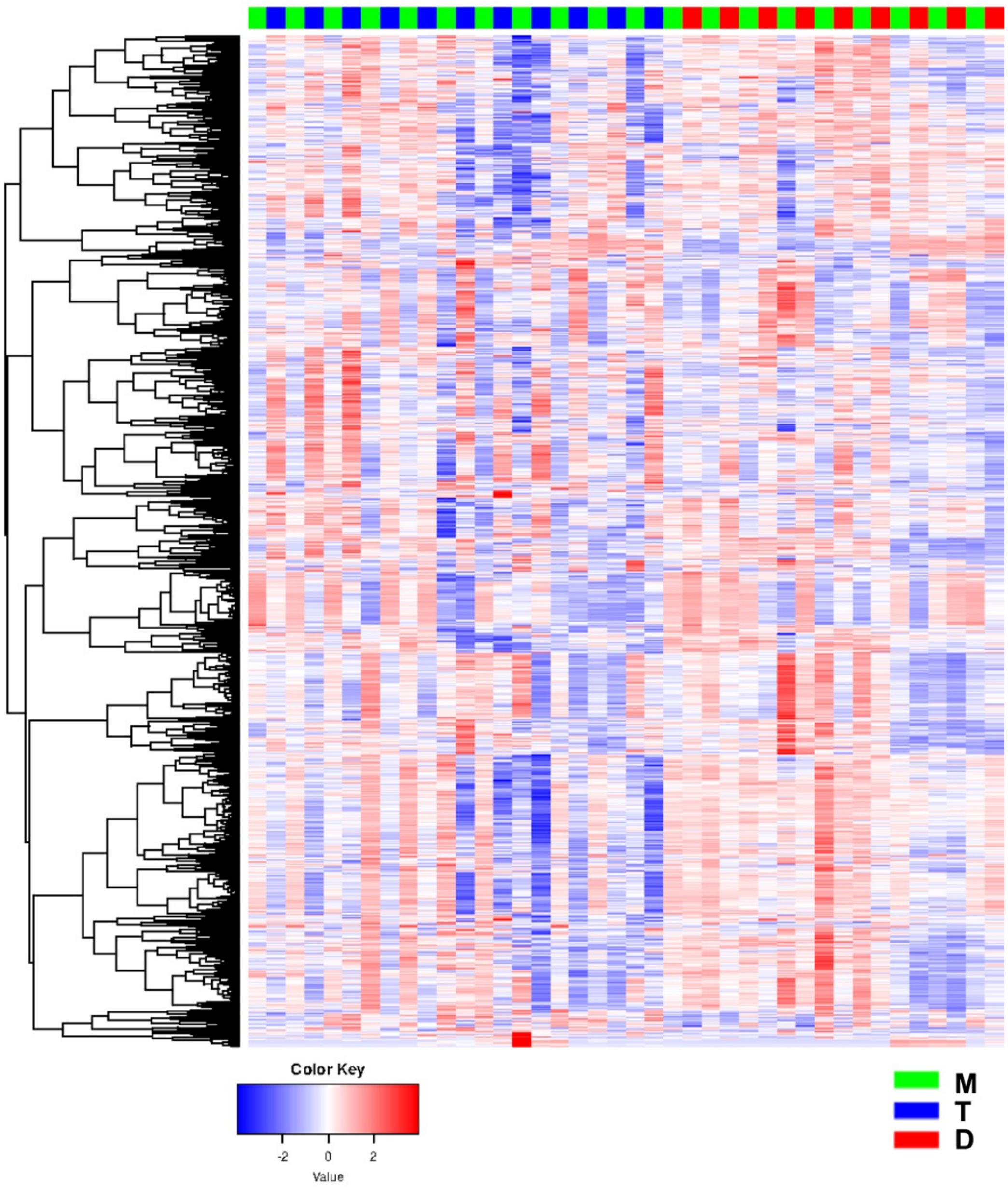

Figure S4.

Individual Sample Correlations Based on D/M: Subtype Signature; Spearman

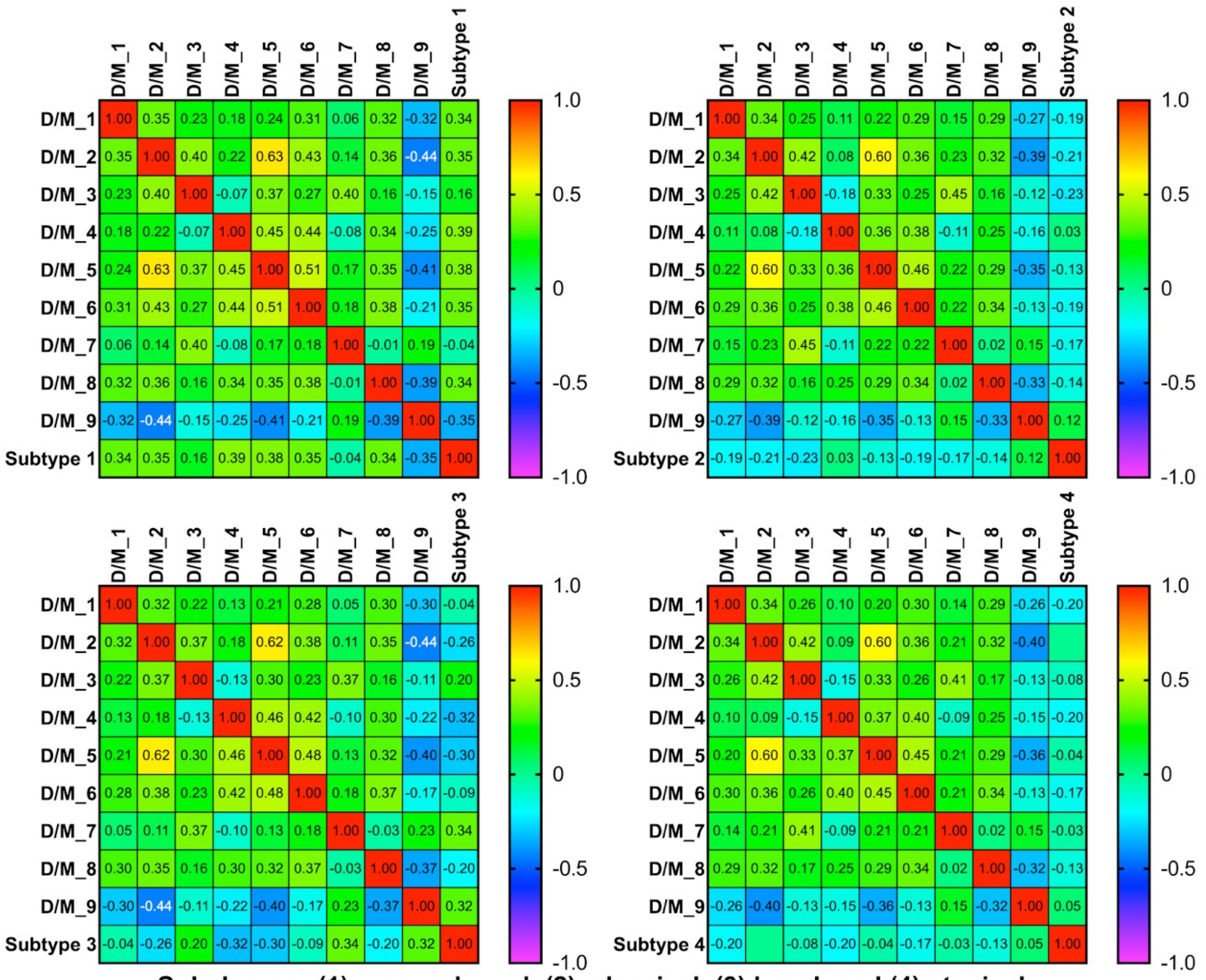

Subclasses: (1) mesenchymal, (2), classical, (3) basal, and (4) atypical

Figure S5A.

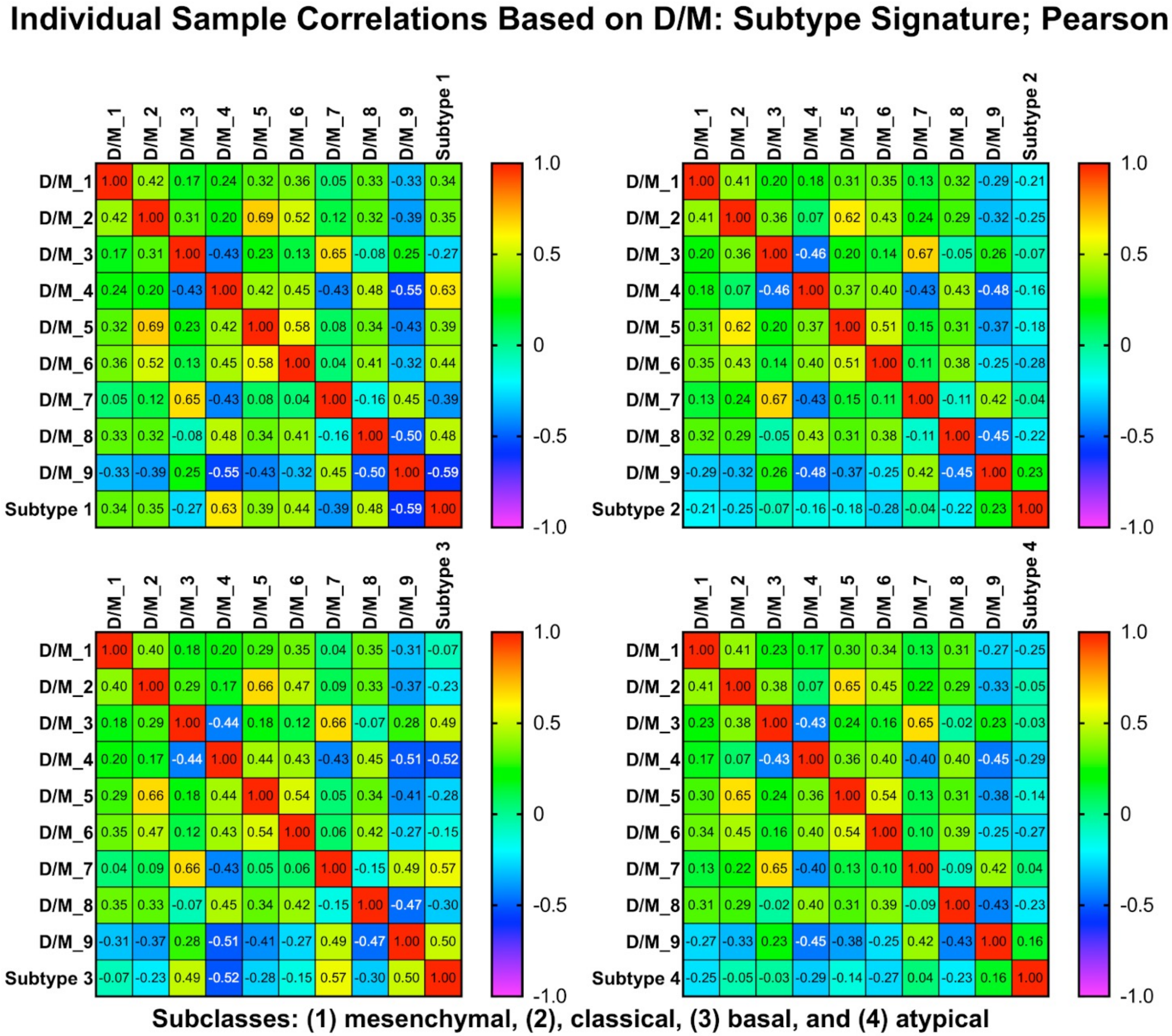
Individual Sample Correlations Based on D/M: Subtype Signature; Pearson
D/M_1 D/M_2 D/M_3 D/M_4 D/M_5 D/M_6 D/M_7 D/M_8 D/M_9 Subtype 1
D/M_1 1.00 0.42 0.17 0.24 0.32 0.36 0.05 0.33 -0.33 0.34
D/M_2 0.42 1.00 0.31 0.20 0.69 0.52 0.12 0.32 -0.39 0.35
D/M_3 0.17 0.31 1.00 -0.43 0.23 0.13 0.65 -0.08 0.25 -0.27
D/M_4 0.24 0.20 -0.43 1.00 0.42 0.45 -0.43 0.48 -0.55 0.63
D/M_5 0.32 0.69 0.23 0.42 1.00 0.58 0.08 0.34 -0.43 0.39
D/M_6 0.36 0.52 0.13 0.45 0.58 1.00 0.04 0.41 -0.32 0.44
D/M_7 0.05 0.12 0.65 -0.43 0.08 0.04 1.00 -0.16 0.45 -0.39
D/M_8 0.33 0.32 -0.08 0.48 0.34 0.41 -0.16 1.00 -0.50 0.48
D/M_9 -0.33 -0.39 0.25 -0.55 -0.43 -0.32 0.45 -0.50 1.00 -0.59
Subtype 1 0.34 0.35 -0.27 0.63 0.39 0.44 -0.39 0.48 -0.59 1.00
1.0 0.5 0 -0.5 -1.0
D/M_1 D/M_2 D/M_3 D/M_4 D/M_5 D/M_6 D/M_7 D/M_8 D/M_9 Subtype 2
D/M_1 1.00 0.41 0.20 0.18 0.31 0.35 0.13 0.32 -0.29 -0.21
D/M_2 0.41 1.00 0.36 0.07 0.62 0.43 0.24 0.29 -0.32 -0.25
D/M_3 0.20 0.36 1.00 -0.46 0.20 0.14 0.67 -0.05 0.26 -0.07
D/M_4 0.18 0.07 -0.46 1.00 0.37 0.40 -0.43 0.43 -0.48 -0.16
D/M_5 0.31 0.62 0.20 0.37 1.00 0.51 0.15 0.31 -0.37 -0.18
D/M_6 0.35 0.43 0.14 0.40 0.51 1.00 0.11 0.38 -0.25 -0.28
D/M_7 0.13 0.24 0.67 -0.43 0.15 0.11 1.00 -0.11 0.42 -0.04
D/M_8 0.32 0.29 -0.05 0.43 0.31 0.38 -0.11 1.00 -0.45 -0.22
D/M_9 -0.29 -0.32 0.26 -0.48 -0.37 -0.25 0.42 -0.45 1.00 0.23
Subtype 2 -0.21 -0.25 -0.07 -0.16 -0.18 -0.28 -0.04 -0.22 0.23 1.00
1.0 0.5 0 -0.5 -1.0
D/M_1 D/M_2 D/M_3 D/M_4 D/M_5 D/M_6 D/M_7 D/M_8 D/M_9 Subtype 3
D/M_1 1.00 0.40 0.18 0.20 0.29 0.35 0.04 0.35 -0.31 -0.07
D/M_2 0.40 1.00 0.29 0.17 0.66 0.47 0.09 0.33 -0.37 -0.23
D/M_3 0.18 0.29 1.00 -0.44 0.18 0.12 0.66 -0.07 0.28 0.49
D/M_4 0.20 0.17 -0.44 1.00 0.44 0.43 -0.43 0.45 -0.51 -0.52
D/M_5 0.29 0.66 0.18 0.44 1.00 0.54 0.05 0.34 -0.41 -0.28
D/M_6 0.35 0.47 0.12 0.43 0.54 1.00 0.06 0.42 -0.27 -0.15
D/M_7 0.04 0.09 0.66 -0.43 0.05 0.06 1.00 -0.15 0.49 0.57
D/M_8 0.35 0.33 -0.07 0.45 0.34 0.42 -0.15 1.00 -0.47 -0.30
D/M_9 -0.31 -0.37 0.28 -0.51 -0.41 -0.27 0.49 -0.47 1.00 0.50
Subtype 3 -0.07 -0.23 0.49 -0.52 -0.28 -0.15 0.57 -0.30 0.50 1.00
1.0 0.5 0 -0.5 -1.0
D/M_1 D/M_2 D/M_3 D/M_4 D/M_5 D/M_6 D/M_7 D/M_8 D/M_9 Subtype 4
D/M_1 1.00 0.41 0.23 0.17 0.30 0.34 0.13 0.31 -0.27 -0.25
D/M_2 0.41 1.00 0.38 0.07 0.65 0.45 0.22 0.29 -0.33 -0.05
D/M_3 0.23 0.38 1.00 -0.43 0.24 0.16 0.65 -0.02 0.23 -0.03
D/M_4 0.17 0.07 -0.43 1.00 0.36 0.40 -0.40 0.40 -0.45 -0.29
D/M_5 0.30 0.65 0.24 0.36 1.00 0.54 0.13 0.31 -0.38 -0.14
D/M_6 0.34 0.45 0.16 0.40 0.54 1.00 0.10 0.39 -0.25 -0.27
D/M_7 0.13 0.22 0.65 -0.40 0.13 0.10 1.00 -0.09 0.42 0.04
D/M_8 0.31 0.29 -0.02 0.40 0.31 0.39 -0.09 1.00 -0.43 -0.23
D/M_9 -0.27 -0.33 0.23 -0.45 -0.38 -0.25 0.42 -0.43 1.00 0.16
Subtype 4 -0.25 -0.05 -0.03 -0.29 -0.14 -0.27 0.04 -0.23 0.16 1.00
1.0 0.5 0 -0.5 -1.0
Subclasses: (1) mesenchymal, (2), classical, (3) basal, and (4) atypical

Figure S5B.

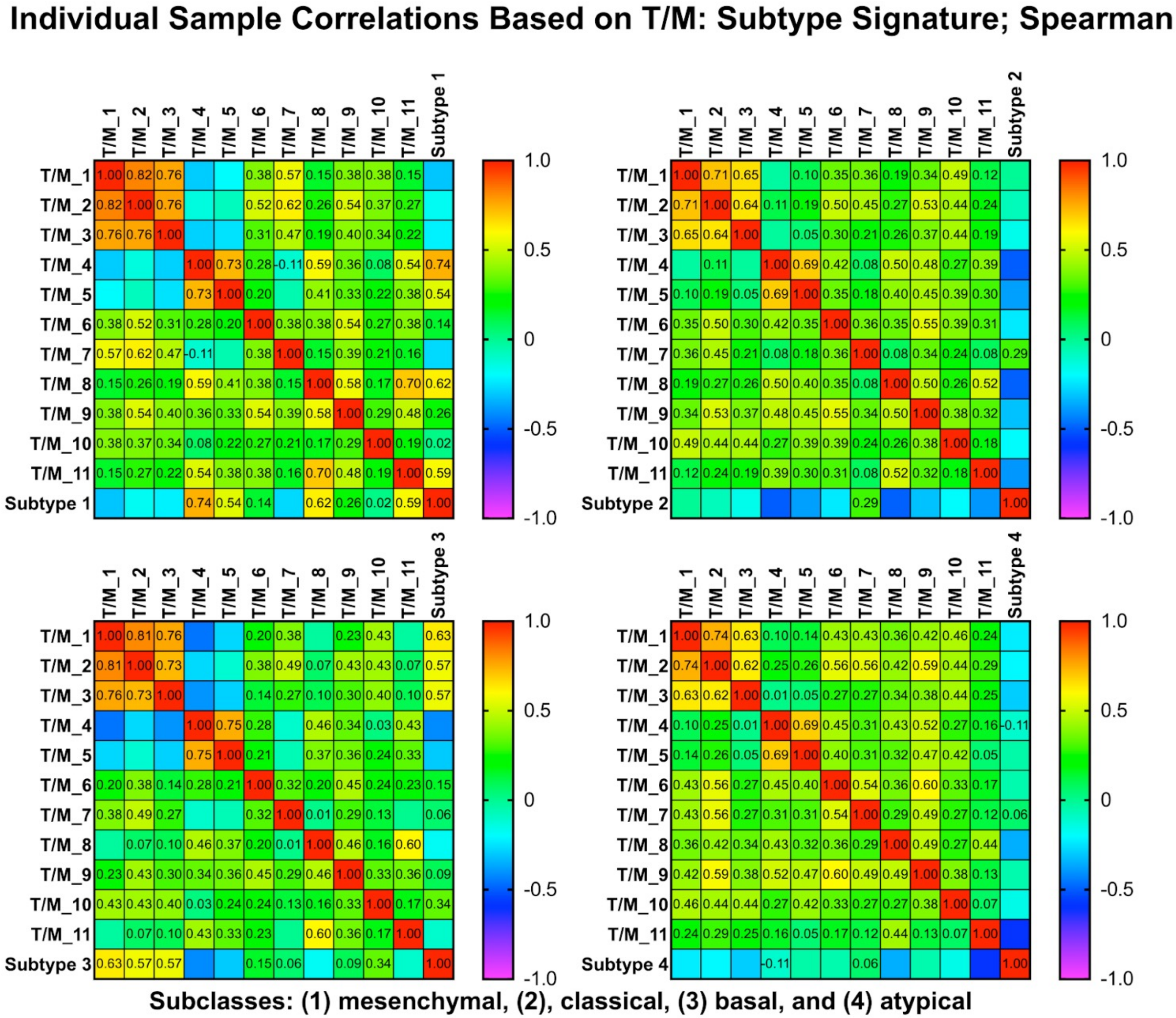
Individual Sample Correlations Based on T/M: Subtype Signature; Spearman
Subclasses: (1) mesenchymal, (2), classical, (3) basal, and (4) atypical

Figure S5C.

Individual Sample Correlations Based on T/M: Subtype Signature; Pearson

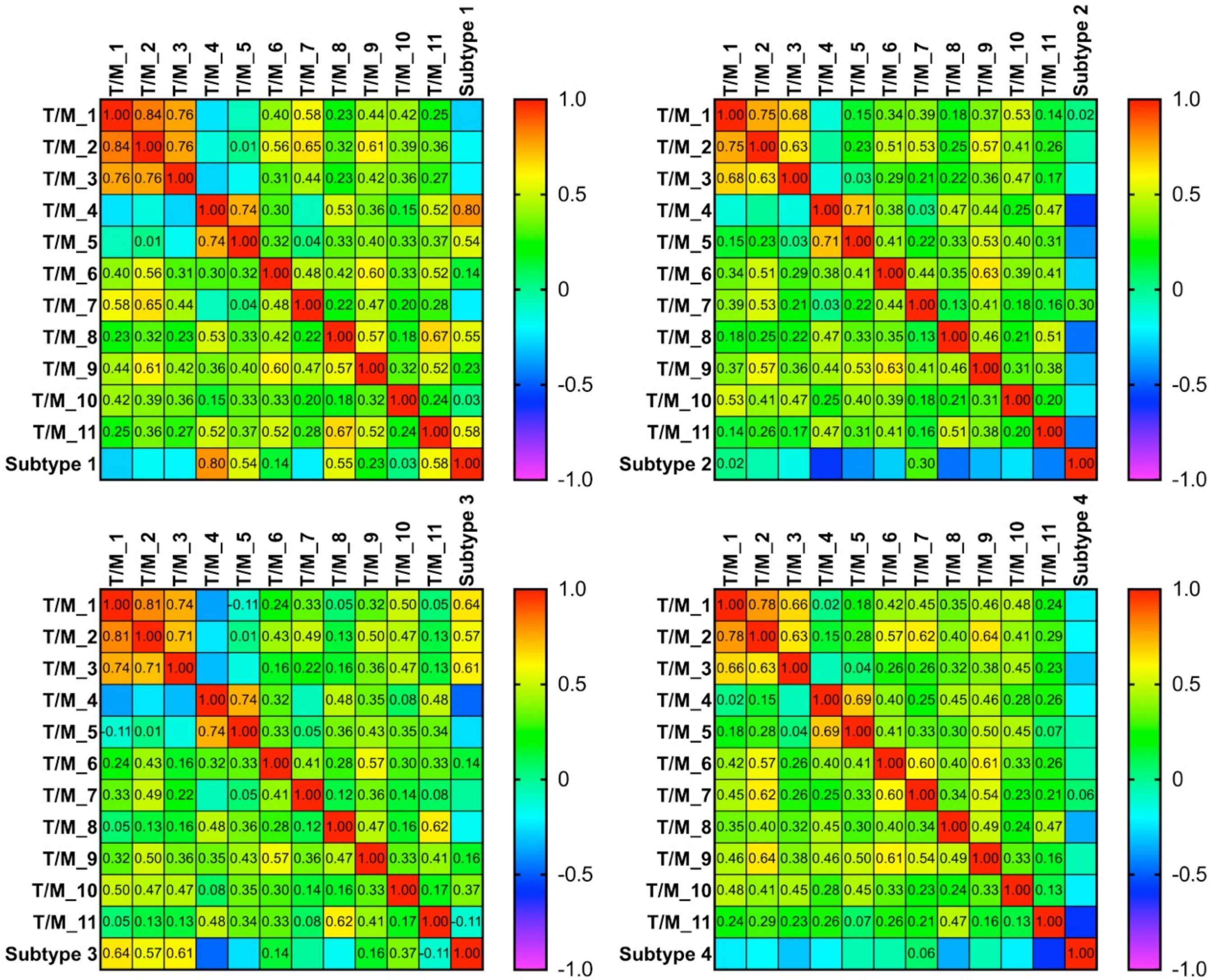

Subclasses: (1) mesenchymal, (2), classical, (3) basal, and (4) atypical

Figure S5D.

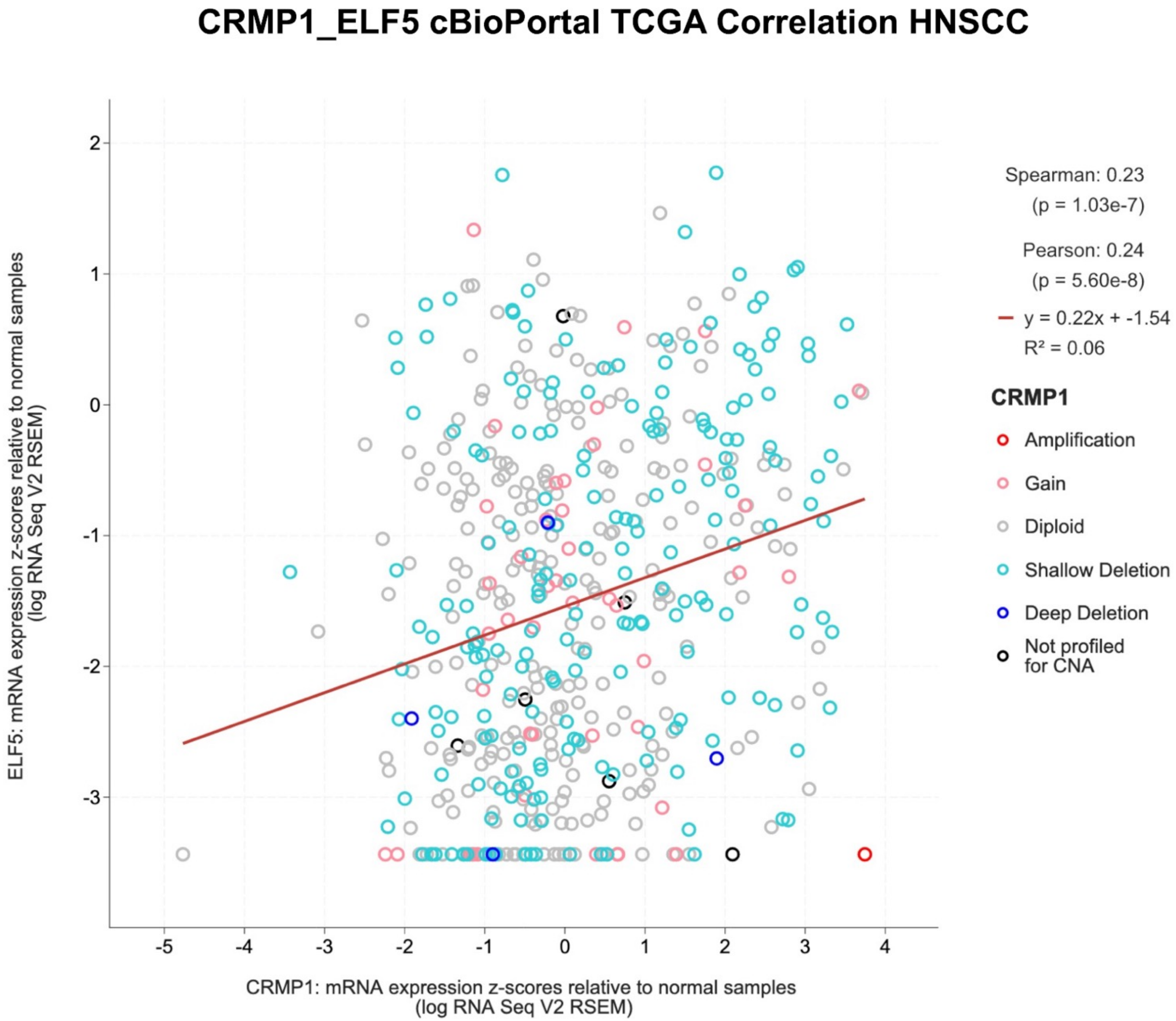
CRMP1_ELF5 cBioPortal TCGA Correlation HNSCC
ELF5: mRNA expression z-scores relative to normal samples
(log RNA Seq V2 RSEM)
CRMP1: mRNA expression z-scores relative to normal samples
(log RNA Seq V2 RSEM)
2
1
0
-1
-2
-3
-5
-4
-3
-2
-1
0
1
2
3
4
Spearman: 0.23
(p = 1.03e-7)
Pearson: 0.24
(p = 5.60e-8)
y = 0.22x + -1.54
R² = 0.06
CRMP1
Amplification
Gain
Diploid
Shallow Deletion
Deep Deletion
Not profiled
for CNA

Figure S6A.

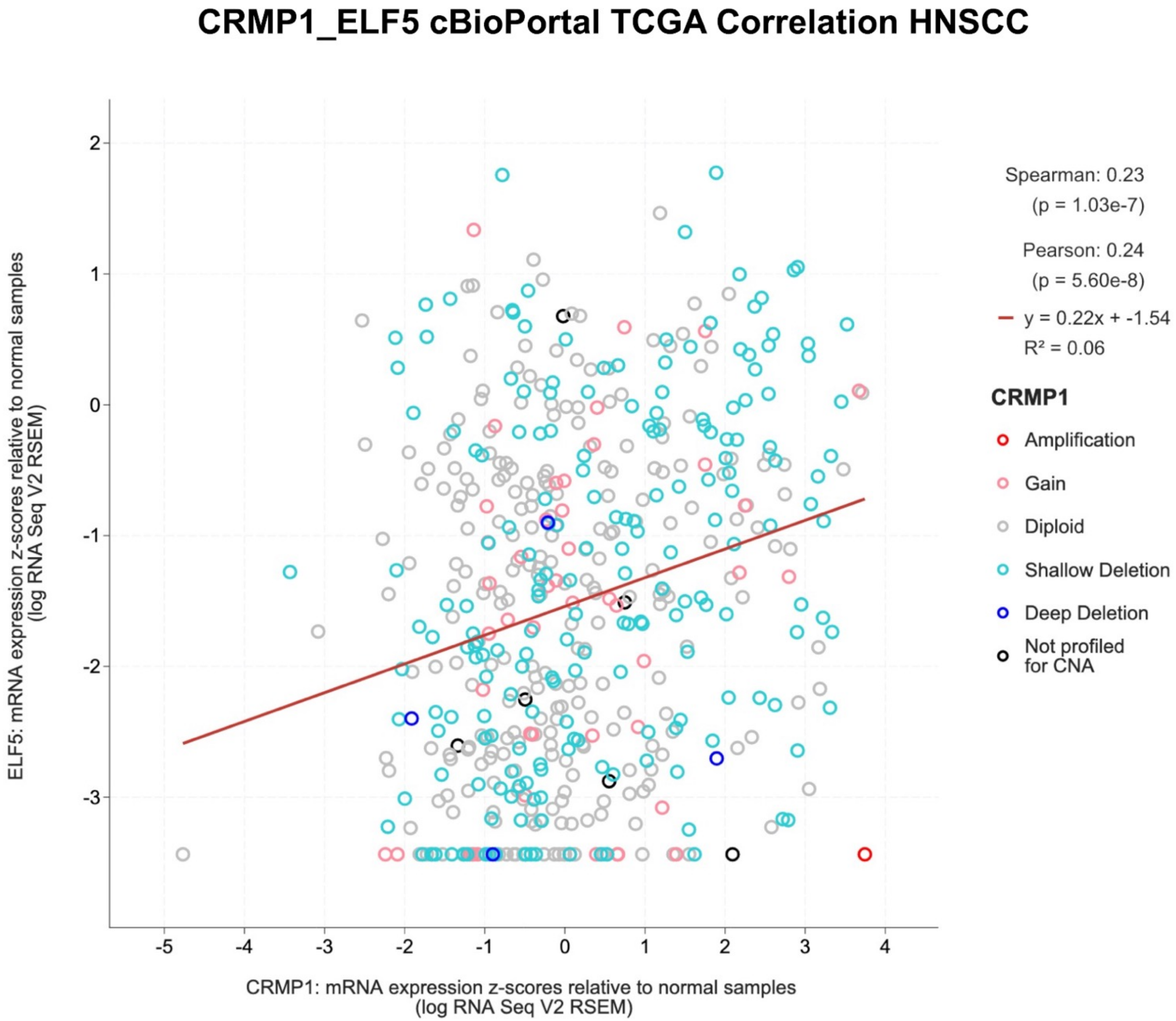

CRMP1_ELF5 cBioPortal TCGA Correlation HNSCC
Spearman: 0.23
(p = 1.03e-7)
Pearson: 0.24
(p = 5.60e-8)
y = 0.22x + -1.54
R² = 0.06
CRMP1
Amplification
Gain
Diploid
Shallow Deletion
Deep Deletion
Not profiled for CNA
ELF5: mRNA expression z-scores relative to normal samples (log RNA Seq V2 RSEM)
CRMP1: mRNA expression z-scores relative to normal samples (log RNA Seq V2 RSEM)
2
1
0
-1
-2
-3
-5
-4
-3
-2
-1
0
1
2
3
4

Figure S6B.

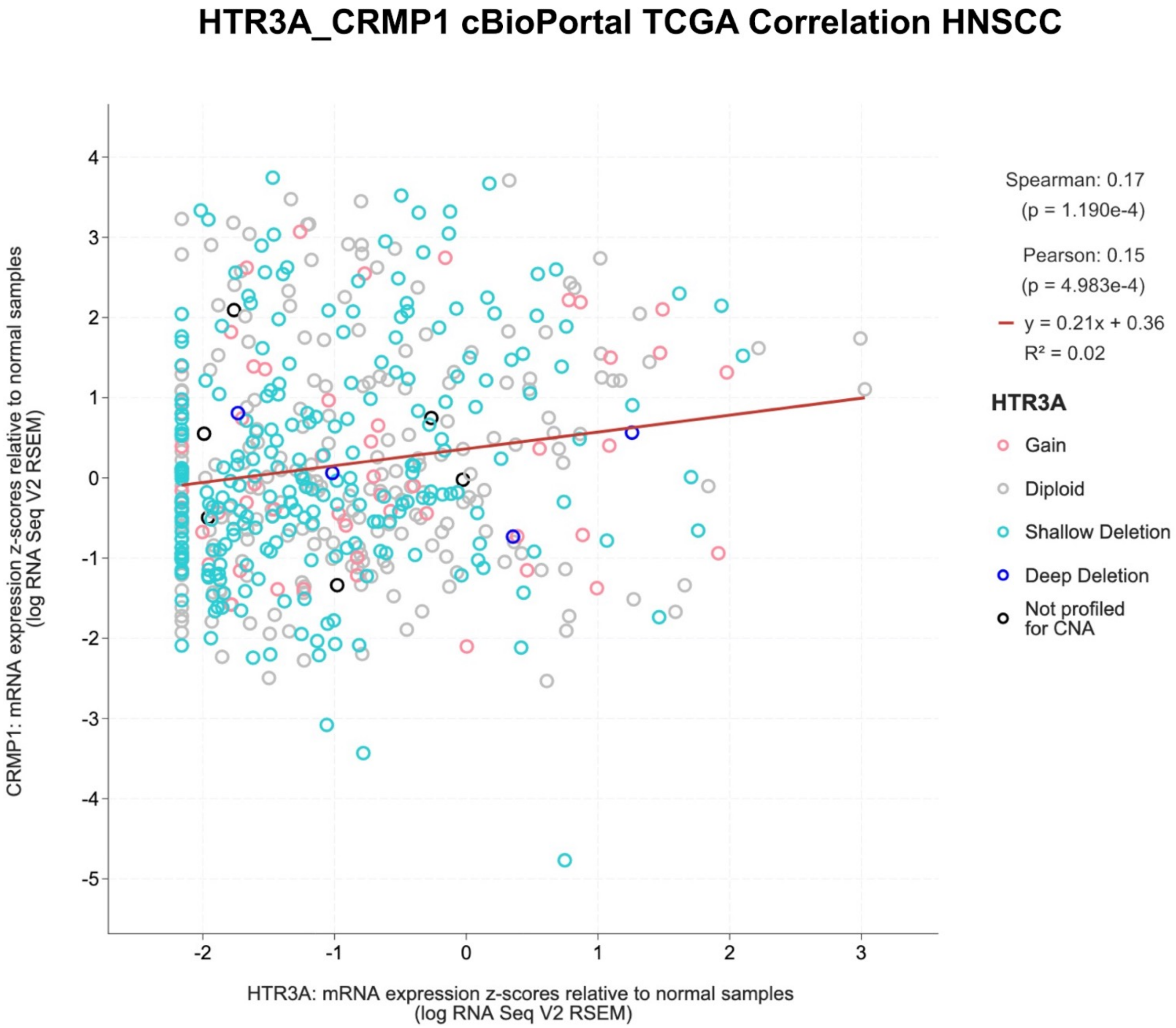

HTR3A_CRMP1 cBioPortal TCGA Correlation HNSCC
Spearman: 0.17
(p = 1.190e-4)
Pearson: 0.15
(p = 4.983e-4)
y = 0.21x + 0.36
R² = 0.02
HTR3A
Gain
Diploid
Shallow Deletion
Deep Deletion
Not profiled for CNA
CRMP1: mRNA expression z-scores relative to normal samples (log RNA Seq V2 RSEM)
HTR3A: mRNA expression z-scores relative to normal samples (log RNA Seq V2 RSEM)
4
3
2
1
0
-1
-2
-3
-4
-5
-2
-1
0
1
2
3

Figure S6C.

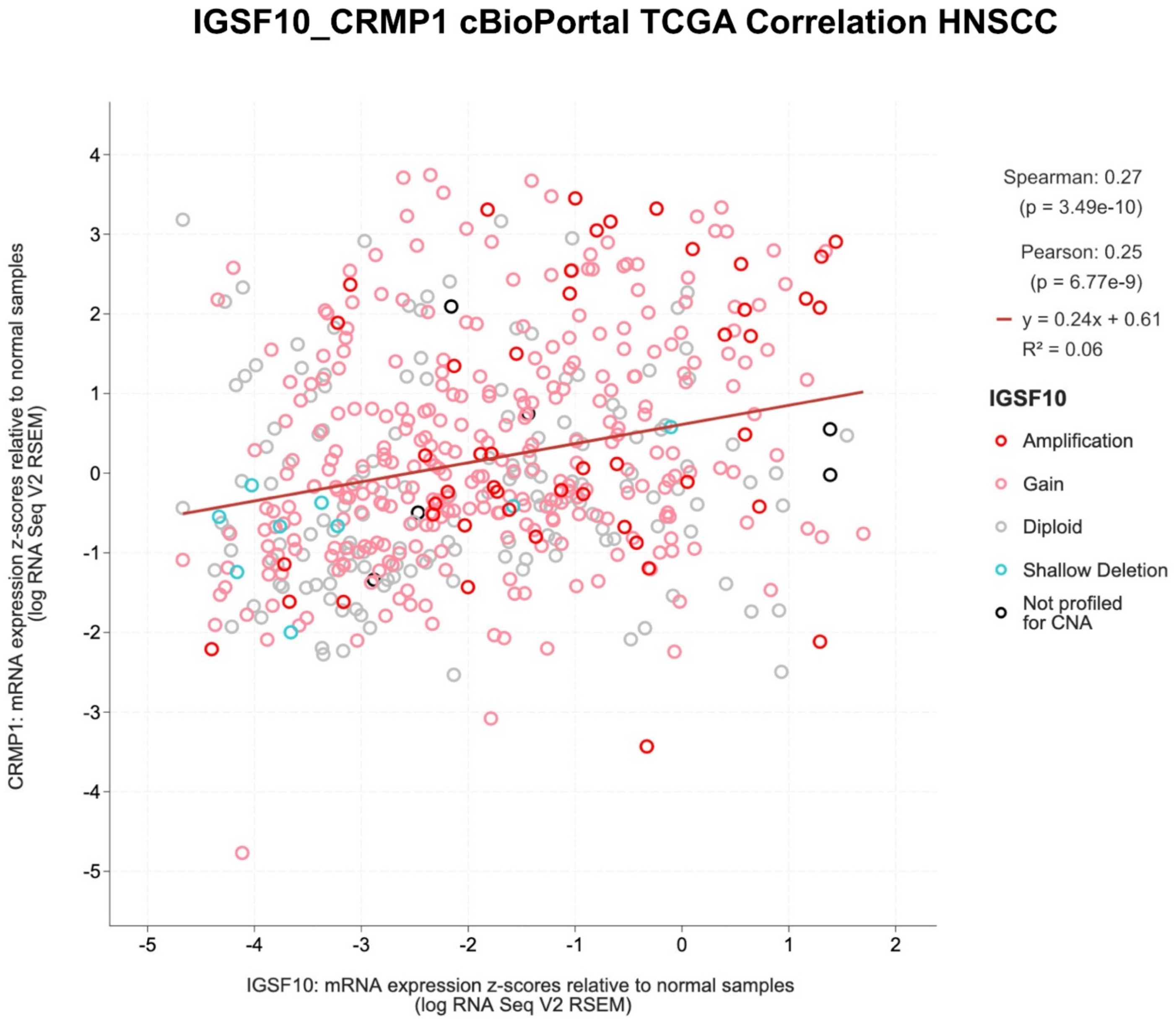

IGSF10_CRMP1 cBioPortal TCGA Correlation HNSCC
CRMP1: mRNA expression z-scores relative to normal samples
(log RNA Seq V2 RSEM)
IGSF10: mRNA expression z-scores relative to normal samples
(log RNA Seq V2 RSEM)
4
3
2
1
0
-1
-2
-3
-4
-5
-5
-4
-3
-2
-1
0
1
2
Spearman: 0.27
(p = 3.49e-10)
Pearson: 0.25
(p = 6.77e-9)
y = 0.24x + 0.61
R² = 0.06
IGSF10
Amplification
Gain
Diploid
Shallow Deletion
Not profiled
for CNA

Figure S6D.

IGSF10_ELF5 cBioPortal TCGA Correlation HNSCC

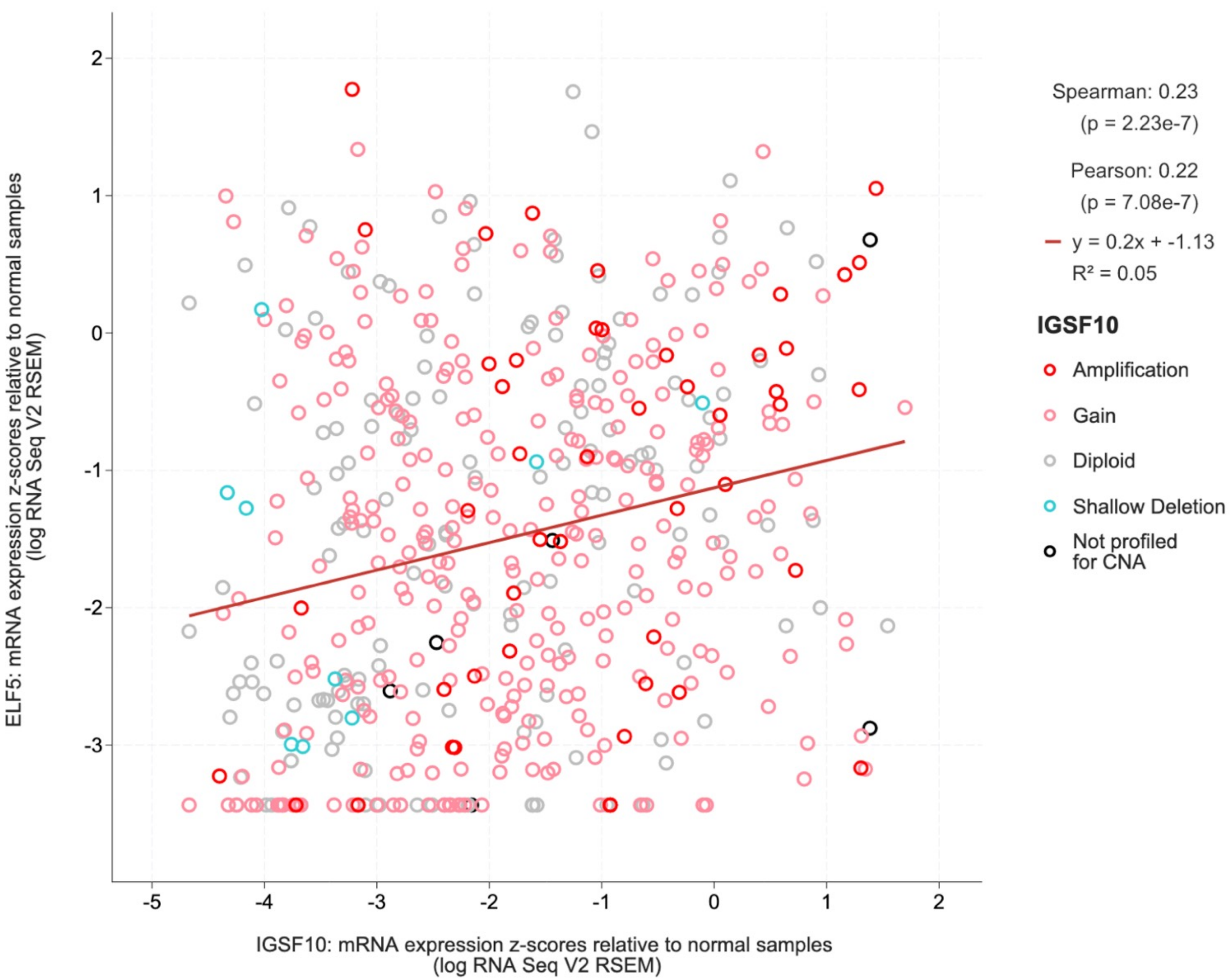

Figure S6E.

IGSF10_HTR3A cBioPortal TCGA Correlation HNSCC

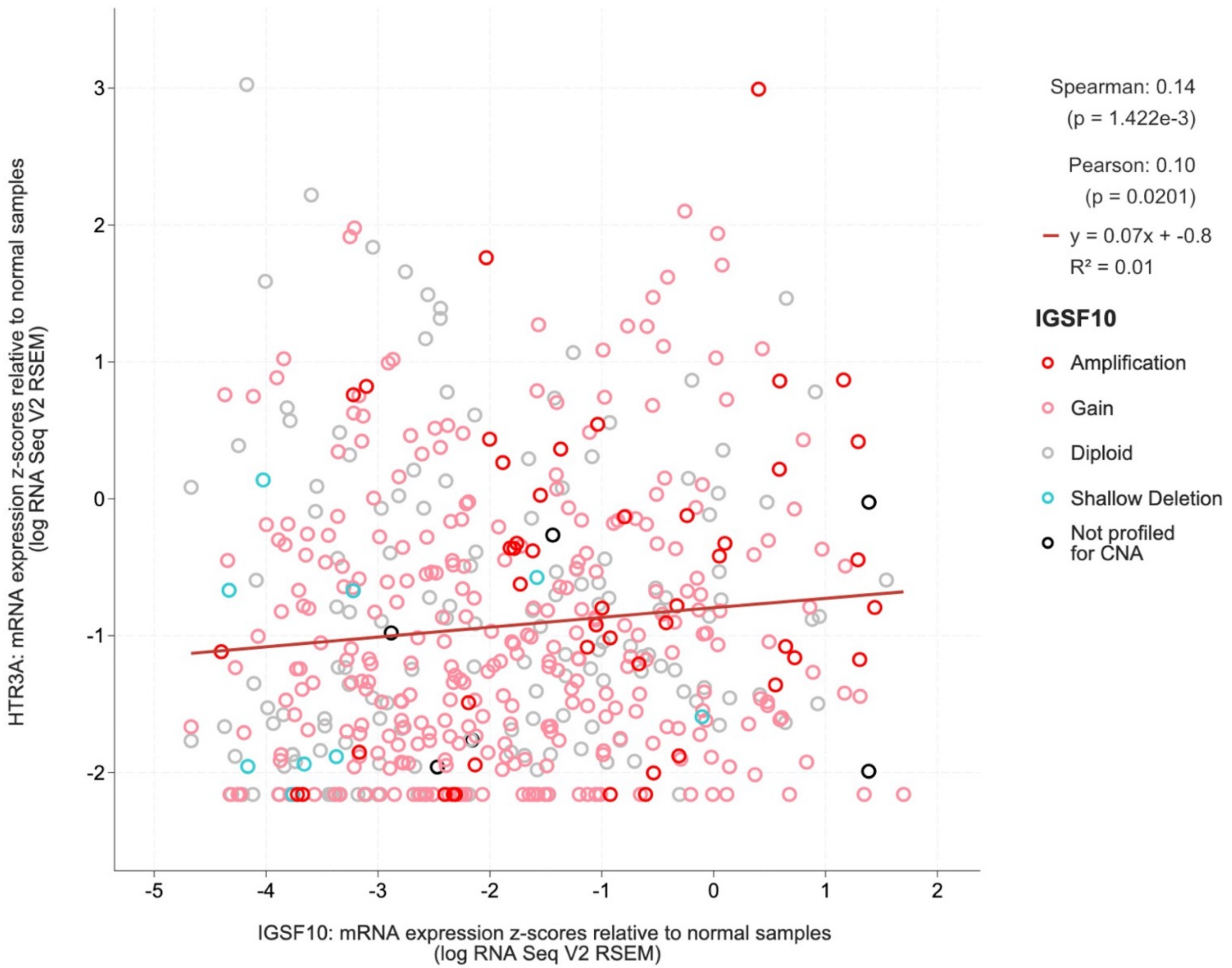

Figure S6F.

IGSF10_RPTN cBioPortal TCGA Correlation HNSCC

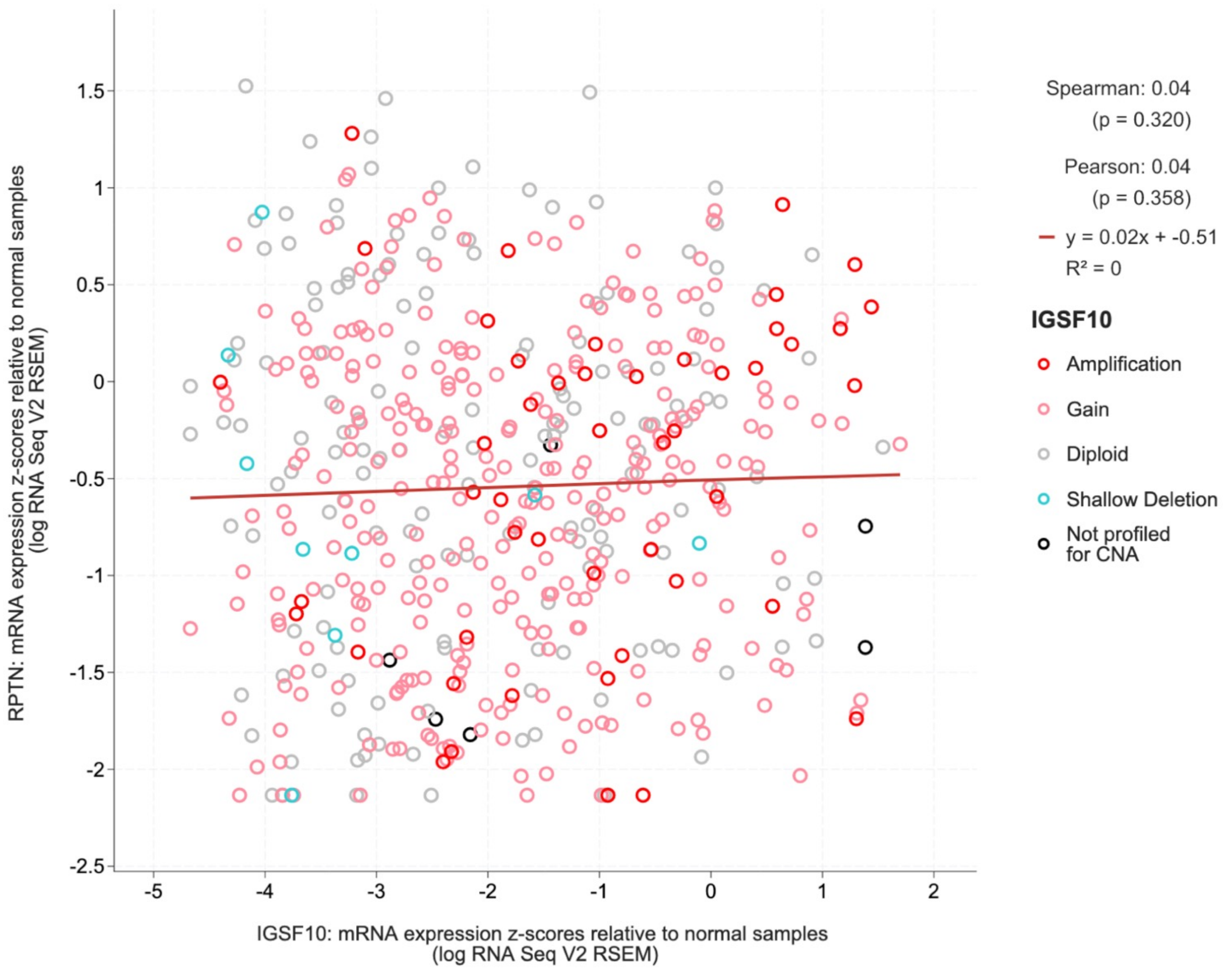

Figure S6G.

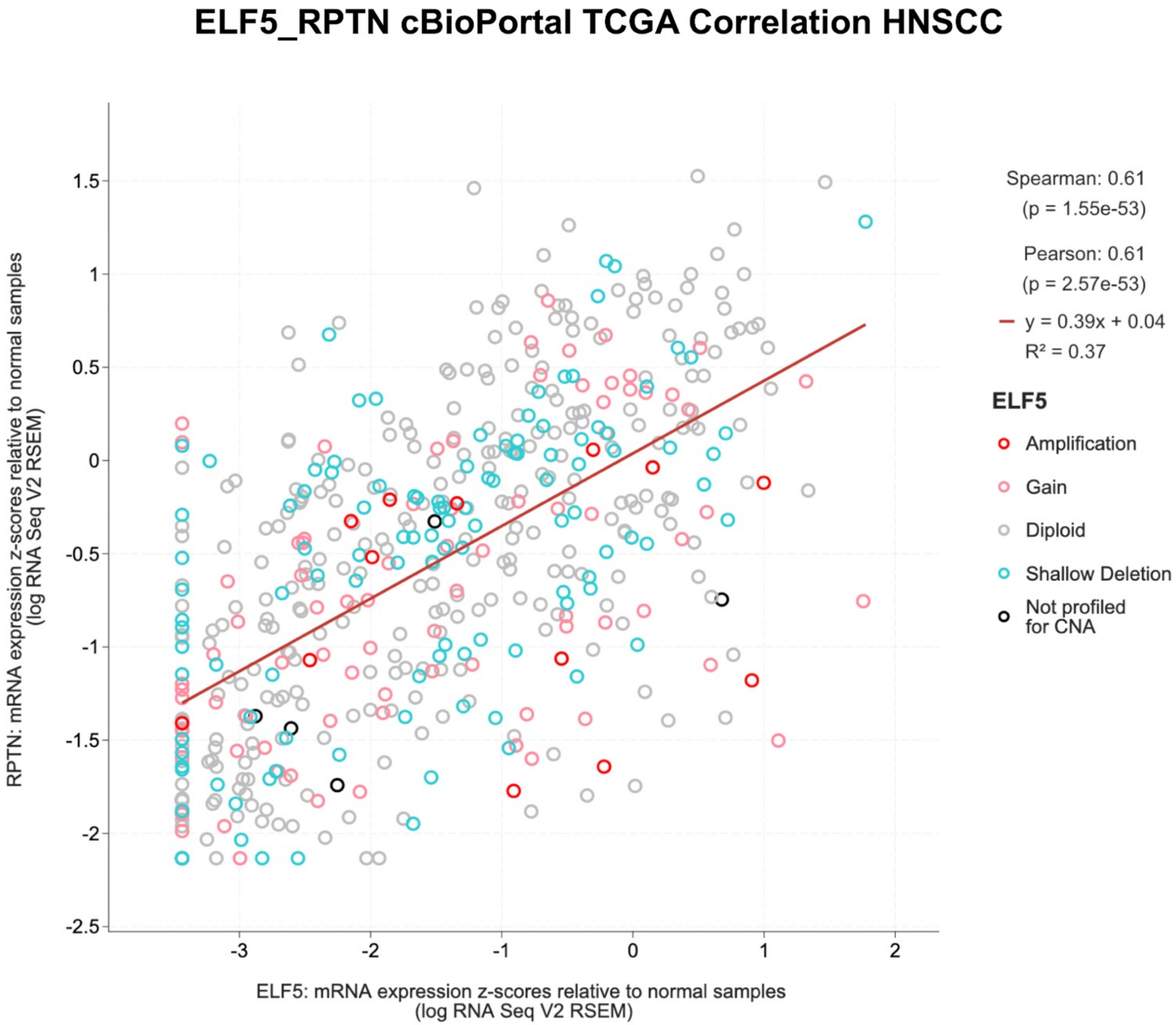

ELF5_RPTN cBioPortal TCGA Correlation HNSCC
Spearman: 0.61
(p = 1.55e-53)
Pearson: 0.61
(p = 2.57e-53)
y = 0.39x + 0.04
R² = 0.37
ELF5
Amplification
Gain
Diploid
Shallow Deletion
Not profiled for CNA
RPTN: mRNA expression z-scores relative to normal samples (log RNA Seq V2 RSEM)
ELF5: mRNA expression z-scores relative to normal samples (log RNA Seq V2 RSEM)
1.5
1
0.5
0
-0.5
-1
-1.5
-2
-2.5
-3
-2
-1
0
1
2

Figure S6H.

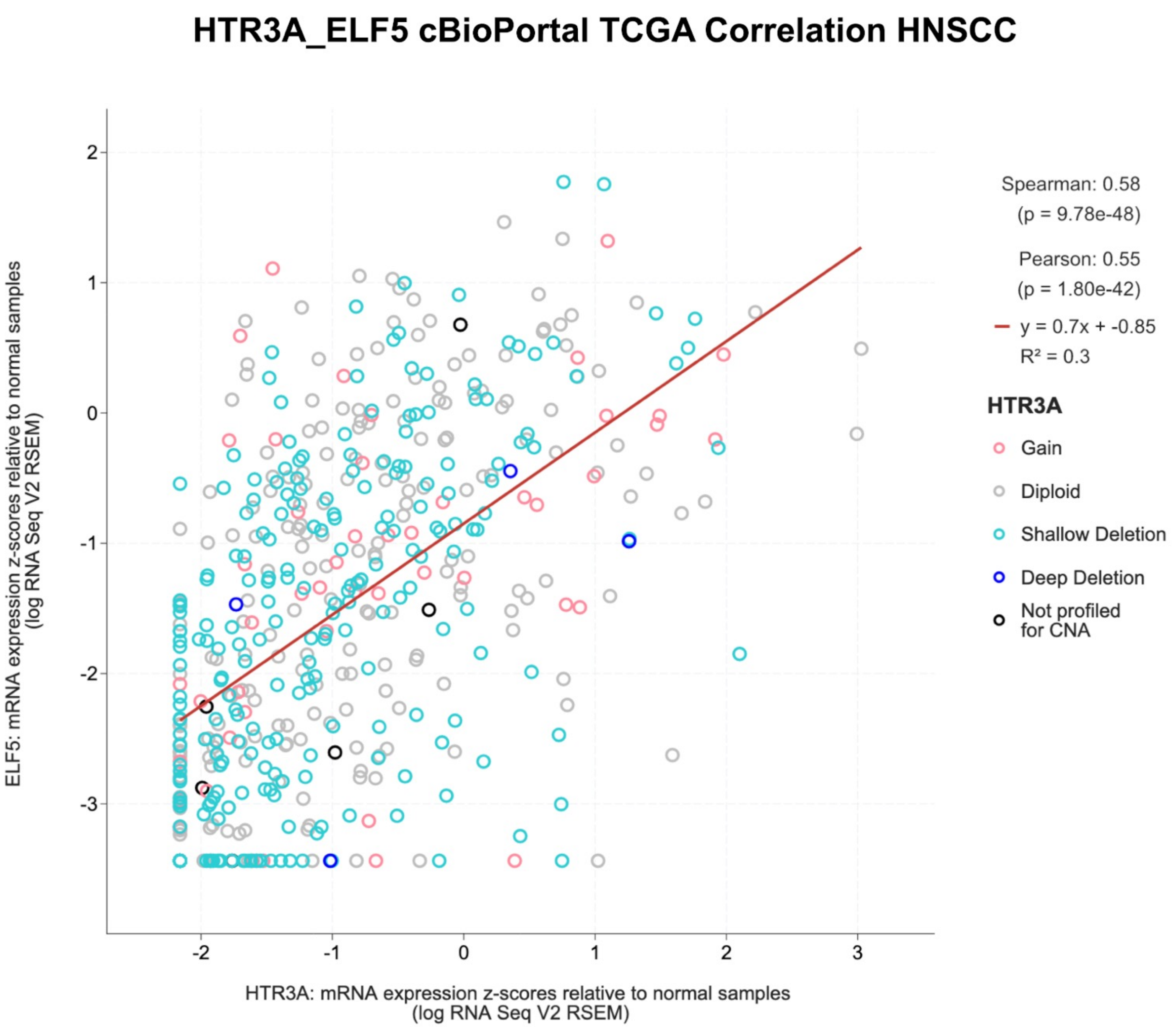
HTR3A_ELF5 cBioPortal TCGA Correlation HNSCC
Spearman: 0.58
(p = 9.78e-48)
Pearson: 0.55
(p = 1.80e-42)
y = 0.7x + -0.85
R² = 0.3
HTR3A
Gain
Diploid
Shallow Deletion
Deep Deletion
Not profiled for CNA
ELF5: mRNA expression z-scores relative to normal samples (log RNA Seq V2 RSEM)
HTR3A: mRNA expression z-scores relative to normal samples (log RNA Seq V2 RSEM)
2
1
0
-1
-2
-3
-2
-1
0
1
2
3

Figure S6I.

HTR3A_RPTN cBioPortal TCGA Correlation HNSCC

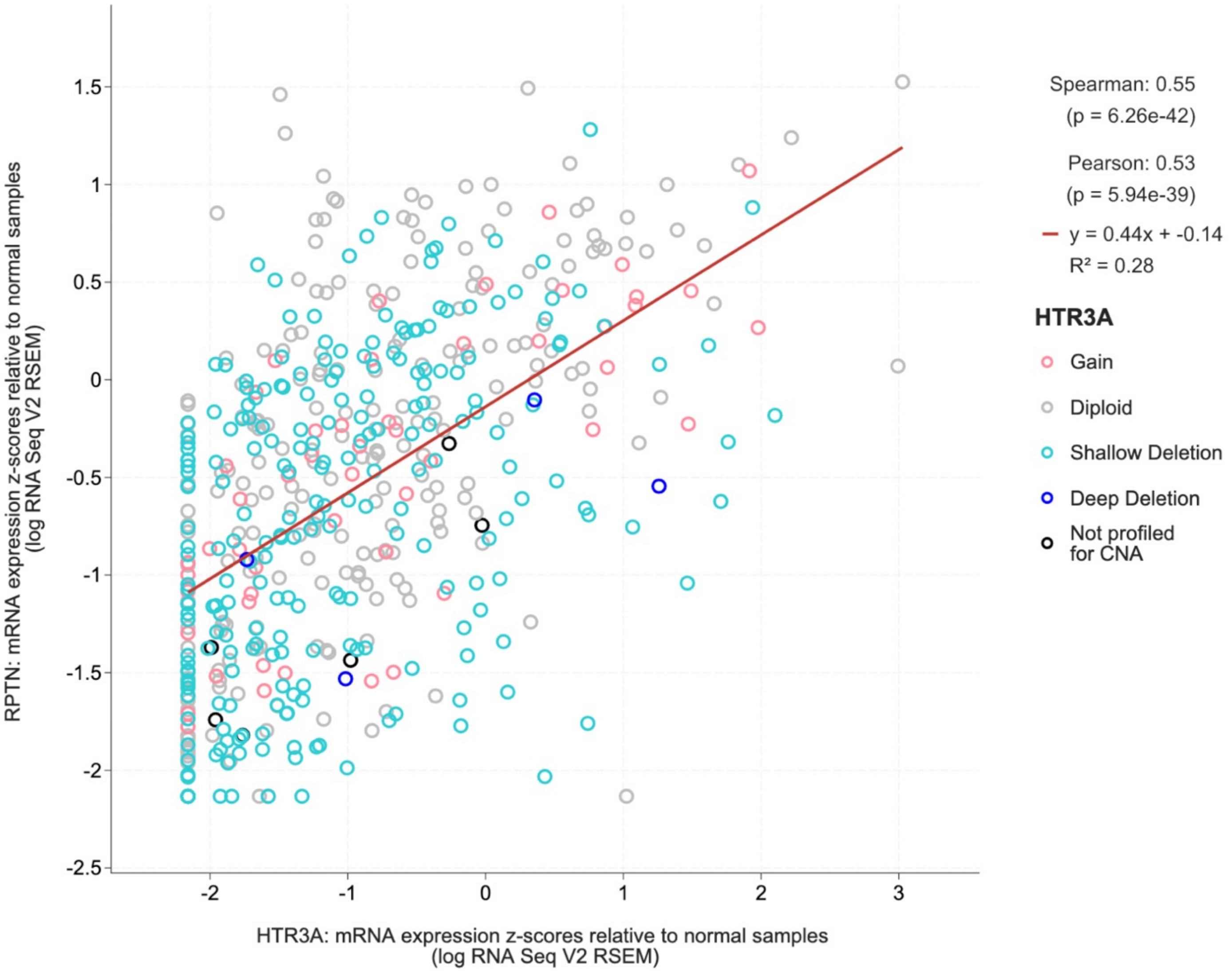

Figure S6J.
