# Supplementary material for: Transcriptomic analysis predicts the risk of progression of premalignant lesions in human tongue
Source: Discov Oncol. 2023 Feb 23;14:24. doi: 10.1007/s12672-023-00629-y (PMC9950315; doi:10.1007/s12672-023-00629-y)
Supplement: Supplementary file 2 — (DOCX 33 KB) [file 12672_2023_629_MOESM2_ESM.docx]

**Title:** Transcriptomic analysis predicts the risk of progression of premalignant lesions in human tongue

Tuo Zhang ^3#^, David Kutler ^2#^, Theresa Scognamiglio ^4^, Lorraine J. Gudas ^1*^, and Xiao-Han Tang ^1*^

1. Department of Pharmacology, Weill Cornell Medical College of Cornell University, New York, NY 10065

2. Division of Head and Neck Surgery in the Department of Otolaryngology at New York Presbyterian Hospital/Weill Cornell Medical Center, New York, NY 10065

3. Genomics Resources Core Facility, Weill Cornell Medical College of Cornell University, New York, NY 10065

4. Division of Anatomic Pathology, New York Presbyterian Hospital, Department of Pathology and Laboratory Medicine, Weill Cornell Medical College of Cornell University, New York, NY 10065

#, Dr. Zhang and Dr. Kutler contributed to this work equally.

* To whom correspondence should be addressed:

Xiao-Han Tang, Ph.D., Department of Pharmacology, Weill Cornell Medical College of Cornell University, 1300 York Avenue, New York, NY, 10065; telephone: +1-212-746-1139; email: [xit2001@med.cornell.edu](mailto:xit2001@med.cornell.edu)

Lorraine J. Gudas, Ph.D., Department of Pharmacology, Weill Cornell Medical College of Cornell University, 1300 York Avenue, New York, NY, 10065; telephone: +1-212-746-6250; email: [ljgudas@med.cornell.edu](mailto:ljgudas@med.cornell.edu)

Key words: tongue squamous cell carcinoma, tongue oral potentially malignant disorders, head and neck SCC subclass, RNA-seq, logistic regression

FINANCIAL SUPPORT

This research was supported by Weill Cornell Medicine funds.

**Supplementary Information**

**MATERIALS AND METHODS**

**Human oral potentially malignant disorders (OPMD) and squamous cell carcinoma (SCC) samples.** Human patient tongue lesion samples and their corresponding margin samples (normal control tongue samples from the same patients) were surgically resected by Dr. David Kutler, an Associated Professor in the Division of Head and Neck Surgery in the Department of Otolaryngology at Weill Cornell Medicine (WCM). Thirty margin and 37 tongue lesion samples, at different progression stages, were used for this study. All tissue samples collected within 10-15 minutes after the resection were minced and stored in RNAlater at 4 °C overnight, and then stored at -70°C until total RNA extraction. The histopathological assessment was conducted by Dr. Theresa Scognamiglio, a pathologist in the Division of Anatomic Pathology in Department of Pathology and Laboratory Medicine at WCM. All procedures were conducted following informed consent from each patient and adhered to the ethical guidelines of Weill Cornell Medicine. We focused on the paired patient samples, *i.e.* the normal tongue epithelial tissue and the lesion tissue were from the same patient for the comparison. Nine patients were diagnosed as having OPMDs of the tongue, and 11 patients had tongue squamous cell carcinomas (SCC) (see Table below). The main clinical and histopathological characteristics of each patient, including sex, age, characteristics of tumors, regional lymph nodal metastases, and distant metastasis, based on the criteria of the current clinical staging system of OSCCs described in (1), are summarized in Table below.

**Histopathological Characteristics of Patients**

| Site | Sample ID | Sex | Age at Surgery | Pathological Stage | Pathology |
| --- | --- | --- | --- | --- | --- |
| Right Mid-lateral tongue | OPMD 1 | M | 72 |  | Focal moderate epithelial dysplasia |
| Posterior lateral tongue | OPMD 2 | M | 26 |  | Mild to focal moderate epithelial dysplasia |
| Right lateral tongue | OPMD 3 | F | 79 |  | Mild to focal moderate epithelial dysplasia, associated with chronic lichenoid mucositis |
| Posterior left buccal | OPMD 4 | F | 72 |  | Focal mild epithelial dysplasia & lichenoid mucositis |
| Left posterior lateral tongue | OPMD 5 | M | 36 |  | Focal moderate epithelial dysplasia |
| Left anterior lateral tongue | OPMD 6 | F | 22 |  | Hyperplastic parakeratotic squamous mucosa with mild epithelial dysplasia |
| Left mid lateral tongue | OPMD 7 | F | 80 |  | Mild epithelial dysplasia |
| Left lateral tongue | OPMD 8 | F | 67 |  | Hyperplastic squamous mucosa with mild hyperparakeratosis, focally associated with lichenoid mucositis |
| Left lateral tongue | OPMD 9 | F | 80 |  | Focal mild epithelial dysplasia |
|  |  |  |  |  |  |
| Right lateral tongue | SCC 1 | M | 79 | T1NxMx |  |
| Left lateral tongue | SCC 2 | M | 68 | T1NxMx |  |
| Right lateral tongue | SCC 3 | M | 19 | T3N0Mx |  |
| Left lateral tongue | SCC 4 | M | 89 | TisN0Mx |  |
| Right poaterior tongue | SCC 5 | M | 66 | Severe keratinzing dysplasia | In-situ and invasive squamous cell carcinoma, well differentiated |
| Left lateral tongue | SCC 6 | M | 67 | T2N0Mx |  |
| Right posterior tongue | SCC 7 | F | 51 | T3N1Mx |  |
| Right posterior tongue | SCC 8 | M | 55 | T2N0Mx |  |
| Left lateral tongue | SCC 9 | F | 70 | T3N1Mx |  |
| Left lateral tongue | SCC 10 | M | 79 | TisN0Mx |  |
| Right lateral tongue | SCC 11 | M | 72 | T3N3bMx |  |

Tis, tumor in situ, i.e., tumor is only in the very top layer of the tongue epithelium.

**Pathological Diagnosis.** Histopathologic examination was performed for all lesions. The lesions were categorized as hyperplasia, epithelial dysplasia or carcinoma. Epithelial dysplasia was graded as mild, moderate, and severe based on the evaluation of both cytologic and architectural atypia. Cytologic abnormalities include nuclear pleomorphism, nuclear hyperchromasia and irregularity, increased nuclear/cytoplasmic ratio, increased mitoses including atypical mitoses (often seen above the basal zone), apoptosis, and dyskeratosis. Architectural abnormalities include loss of polarity and irregular distribution of cells, elongated rete ridges (bulbous, drop shaped), and loss of maturation with increased cellularity. Carcinoma was categorized as either in-situ or invasive.

**RNA-seq analysis of transcriptome**.

***Total RNA preparation.*** We prepared total RNA from the human margin, OPMD, and SCC samples using the RNeasy kit (Qiagen). Subsequent steps were carried out at the Genomics Resources Core Facility of WCMC. RNA integrity was measured using the Agilent 2100 BioAnalyzer (Agilent Technologies).

Subsequent RNA preparation steps were carried out at the Genomics Resources Core Facility of WCM. Samples with RNA integrity number (RIN) values of 8.5 and above were used to construct cDNA libraries using the Illumina Stranded mRNA Prep kit. The sequencing was conducted with pair-end 51 bases on the Illumina NovaSeq 6000.

***cDNA library construction.*** Samples with RNA integrity number (RIN) values of >9 were used to construct cDNA libraries. cDNA synthesis, end-repair, and ligation to the Illumina indexed adapters were performed from the RNA samples by the TruSeq RNA protocol (Illumina). Libraries of 250-300 bp cDNA sizes were PCR-amplified using Phusion DNA polymerase. Following the removal of mRNA strands by RNaseH, first strand cDNAs were used as templates to produce double strand cDNAs. The overhangs resulting from fragmentation were repaired to blunt ends. An ‘A’ base was added to the 3’ end of cDNAs and subsequently the cDNAs were ligated to Illumina paired end (PE) adaptors that have a single ‘T’ base overhang at their 3' end. The cDNA-adaptor libraries were purified and enriched by 15 cycles of PCR. The enriched libraries were hybridized to a flow cell and amplified, resulting in ultra-high density flow cells with millions of clusters, each containing about 1,000 copies of the templates. The double stranded cDNA-adaptors were denatured and converted into single strand DNA, and then the template cDNAs were amplified one more time isothermally to produce surface-bound colonies. The clonal DNA clusters were linearized, free 3’ OH ends blocked, denatured, hybridized to sequencing primers.

***cDNA library sequencing and data analysis.***

The libraries were sequenced on the Illumina HiSeq 4000 with paired-end 51 bps and ~30 million paired reads per sample. The Sequencing-by-Synthesis process used reversible terminators and a DNA polymerase modified to accept reversible terminator nucleotides. After each synthesis cycle the fluorescence of clusters was imaged with high sensitivity. Then the sequencing images were analyzed in three steps, image analysis, base calling, and sequence analysis. Paired-end transcriptome sequencing reads were aligned to the reference mouse genome (UCSC mm9 assembly) using Tophat v2.0.11. Raw read counts were calculated using HTseq-count (2). DESeq2 v1.6.3 was used to perform differential expression analysis, principal component analyses and sample clustering. The removeBatchEffect function from the limma package v3.22.7 was used to remove batch effects introduced in processing samples from different batches. For differential expression analysis, pairwise comparisons between two or more groups using parametric tests where read-counts follow a negative binomial distribution with a gene-specific dispersion parameter. Corrected p-values were calculated based on the Benjamini-Hochberg method to be adjusted for multiple testing. The heatmaps for genes of interest were generated by R pheatmap package software.

***RNA-seq data analysis.***

We used FastQC (v0.11.15) ( <http://www.bioinformatics.babraham.ac.uk/projects/fastqc/>) to perform QC on the raw sequencing reads, and used cutadapt (v1.9.1) to trim adapter sequences and low quality bases in the reads. We used Tophat2 (v2.0.11) (3) to align the cleaned reads against the human hg19 reference genome and Cufflinks (v 2.1.1) (4) to measure transcript abundances in the unit of fragments per kilobases of transcript per million mapped reads (FPKM). We calculated the raw read counts per gene using the HTSeq-count (v0.11.2) and performed differential expression analysis for OPMD and SCC samples separately using DESeq2 package (v 1.26.0) (5). We applied a multi-factor design, by accounting for the normal-lesion sample pairing, to retrieve expression changes attributable to the lesion rather than variations of individuals. We applied a regularized log transformation on the read counts and used the removeBatchEffect function from R limma package (v 3.42.0) to remove individual-specific effects. The heatmaps and PCA plots for global transcriptome were generated by R package software and iDEP (integrated Differential Expression and Pathway analysis) (<http://bioinformatics.sdstate.edu/idep/>) (6).

**Pathway and gene ontology analysis.** We used Enrichr (<https://maayanlab.cloud/Enrichr/>) (7) for pathway and gene ontology analysis. The databases used were "Disease Perturbations from GEO Down”, "Disease Perturbations from GEO Up”; MSigDB (The Molecular Signatures Database) (MSigDB v7.4, released in 2021); KEGG (Kyoto Encyclopedia of Genes and Genomes); and GO-BP (The Gene Ontology-Biological Process).

**SCC subclass correlation studies.** We carried out Pearson and Spearman correlation studies using GraphPad Prism software. Transcript levels in OPMD and SCC samples that varied by at least two-fold (Log2>1 or Log2<-1, relative to the corresponding margin tissue) were used in these studies.

**Firth logistic regression analysis.** Our RNA-seq data show that the changes in transcripts in OPMD and SCC samples produced a numeric value on a continuous scale, not a binary pattern, suggesting that we could not directly use the transcript level changes for this prediction. To alleviate possible overfitting due to small sample sizes, we built a logistic regression model to classify OPMD and SCC samples based on gene expression changes (log2 fold change).

We pre-selected genes that are differentially expressed in either OPMD or SCC groups with adjusted p-value (q value) < 0.05. We ranked these genes based on their power in separating OPMD/SCC from normal condition. Specifically, we built Firth logistic regression model using one gene at a time and evaluated its prediction power using AUC. This process was done using “Leave-One-Out” cross validation (LOOCV) to minimizing overfitting, i.e., we trained a model using 19 samples and tested it on the one left-out sample, repeating this procedure 20 times so that all samples were used exactly once for testing. We then evaluated its power in distinguishing between OPMD and SCC groups with the area under curve (AUC) of the Receiver Operator Characteristic (ROC) curve. We further screened a set of genes with the following criteria:

1) Adjusted p-value (padj) filter (significant in both OPMDs and SCCs): padj < 0.05 for both OPMD and SCC

2) Log2 fold change (FC) filter (opposite directions in OPMD and SCC): log2FC < -0.5 for OPMD and log2FC > 0.5 for SCC or vice versa

3) BaseMean filter (with certain level of expressions): baseMean > 50 for both OPMD and SCC

4) AUC filter (power to separate OPMD from SCC): AUC > 0.8

We ran a greedy search algorithm (8) on all possible combinations of the genes that passed these filters with LOOCV; we selected the gene combinations of the highest AUCs (AUC=1) to build the final prediction models. The Firth logistic regression analysis was implemented using the R logistf package (v1.24.1). The AUC was calculated using the R ROCR package (v1.0_11).

During the process of training and evaluating a prediction model, we applied LOOCV to obtain an unbiased assessment of how well our prediction model performs on new data.

**Data and code availability.** The data reported in this paper have been deposited in the Gene Expression Omnibus (GEO) database, www.ncbi.nlm.nih.gov/geo. The Gene Expression Omnibus accession number is GSE202048, embargoed until publication. A web application to predict the SCC risk of a tongue OPMD is available at <https://freshtuo.shinyapps.io/sccpred/>. The code used to train and test our prediction model is available upon request.

**Figure S1.** **The common transcripts with significantly altered levels (q<0.05, log2>1 or <-1) between the OMD and SCC samples.** **(A)** Venn diagram showing the overlap of transcripts altered in OPMD and SCC. **(B)** Fisher Exact Test proving that this overlap in **(A)** is not expected random chances. **(C)** Log2 fold changes of the common 146 transcripts in both OPMD and SCC. **(D)** Heatmap comparing the log2 fold changes of these common 146 transcripts in OPMD/margin and SCC/margin of individual patients. D (disorders), OPMD, D/M, OPMD/margin; T, tumor, T/M, SCC/margin.

**Figure S2. KEGG (Kyoto Encyclopedia of Genes and Genomes) pathway analysis of transcriptomic changes (q<0.05, log2>1 or <-1) in human tongue oral potentially malignant disorders (OPMD) and squamous cell carcinoma (SCC).** Left Y axis, odds ratio; right Y axis, -Log10 q-value.

**Figure S3. Enrichment analysis of altered transcripts (q<0.05, log2>1 or <-1) using Gene Ontology (GO) Biological Process Database.** Left Y axis, odds ratio; right Y axis, -Log10 q-value.

**Figure S4. Heatmap showing global transcriptomic profiles of individual margin, OPMD, and SCC samples.**  Each margin sample is followed by an OPMD or SCC sample from the same patient. D (disorders), OPMD; M, margin; T (tumor), SCC.

**Figure S5 Human head and neck squamous cell carcinoma (HNSCC) subclass categorization of the OPMD and SCC samples, based on the global transcriptomic changes (q<0.05, log2>1 or <-1) in individual OPMD or SCC.**  Heatmaps showing the association among each OPMD and SCC samples and their correlations with each human head and neck squamous cell carcinoma (SCC) subclass. D (disorders), OPMD; M, margin; T (tumor), SCC; D/M, OPMD/margin; T/M, SCC/margin.

**Figure S6 Pearson and Spearman correlation studies among the changes in the transcripts of ELF5, RPTN, IGSF10, CRMP1, HTR3A within the OPMD and SCC groups using human head and neck squamous cell carcinoma (HNSCC) TCGA PanCancer Atlas database.**

1. Edge SB, Compton CC. The American Joint Committee on Cancer: the 7th edition of the AJCC cancer staging manual and the future of TNM. Ann Surg Oncol **2010**;17:1471-4

2. Anders S, Pyl PT, Huber W. HTSeq--a Python framework to work with high-throughput sequencing data. Bioinformatics **2015**;31:166-9

3. Kim D, Pertea G, Trapnell C, Pimentel H, Kelley R, Salzberg SL. TopHat2: accurate alignment of transcriptomes in the presence of insertions, deletions and gene fusions. Genome Biol **2013**;14:R36

4. Trapnell C, Williams BA, Pertea G, Mortazavi A, Kwan G, van Baren MJ*, et al.* Transcript assembly and quantification by RNA-Seq reveals unannotated transcripts and isoform switching during cell differentiation. Nat Biotechnol **2010**;28:511-5

5. Love MI, Huber W, Anders S. Moderated estimation of fold change and dispersion for RNA-seq data with DESeq2. Genome Biol **2014**;15:550

6. Ge SX, Son EW, Yao R. iDEP: an integrated web application for differential expression and pathway analysis of RNA-Seq data. BMC Bioinformatics **2018**;19:534

7. Kuleshov MV, Jones MR, Rouillard AD, Fernandez NF, Duan Q, Wang Z*, et al.* Enrichr: a comprehensive gene set enrichment analysis web server 2016 update. Nucleic Acids Res **2016**;44:W90-7

8. Mitra K, Carvunis AR, Ramesh SK, Ideker T. Integrative approaches for finding modular structure in biological networks. Nat Rev Genet **2013**;14:719-32

9. Valastyan S, Weinberg RA. Tumor metastasis: molecular insights and evolving paradigms. Cell **2011**;147:275-92
